# Screening of the High-Rhizosphere Competent *Limoniastrum monopetalum*’ Culturable Endophyte Microbiota Allows the Recovery of Multifaceted and Versatile Biocontrol Agents

**DOI:** 10.3390/microorganisms7080249

**Published:** 2019-08-09

**Authors:** Houda Ben Slama, Mohamed Ali Triki, Ali Chenari Bouket, Fedia Ben Mefteh, Faizah N. Alenezi, Lenka Luptakova, Hafsa Cherif-Silini, Armelle Vallat, Tomasz Oszako, Neji Gharsallah, Lassaad Belbahri

**Affiliations:** 1 NextBiotech, 98 Rue Ali Belhouane, Agareb 3030, Tunisia; 2 Institut de l’Olivier Sfax, Sfax 3000, Tunisia; 3 Plant Protection Research Department, East Azarbaijan Agricultural and Natural Resources Research and Education Center, AREEO, 5355179854 Tabriz, Iran; 4 Faculty of Science, B.P. 1171, 3000, University of Sfax, Sfax 3029, Tunisia; 5 Department of Environmental Technology Management, College of Life Sciences, Kuwait University, P.O. Box 5969, Safat 13060, Kuwait; 6 Department of Biology and Genetics, Institute of Biology, Zoology and Radiobiology, University of Veterinary Medicine and Pharmacy, 04181 Kosice, Slovakia; 7 Laboratory of Applied Microbiology, Department of Microbiology, Faculty of Natural and Life Sciences, University Ferhat Abbas of Setif, 19000 Setif, Algeria; 8 Neuchatel Platform of Analytical Chemistry, Institute of Chemistry, University of Neuchatel, 2000 Neuchatel, Switzerland; 9 Department of Forest Protection, Forest Research Institute, 05-090 Raszyn, Poland; 10 Laboratory of Soil Biology, University of Neuchatel, 2000 Neuchatel, Switzerland

**Keywords:** *Limoniastrum monopetalum*, *Fusarium oxysporum*, biocontrol agents, endophyte, PGPR

## Abstract

Halophyte *Limoniastrum monopetalum*, an evergreen shrub inhabiting the Mediterranean region, has well-documented phytoremediation potential for metal removal from polluted sites. It is also considered to be a medicinal halophyte with potent activity against plant pathogens. Therefore, *L. monopetalum* may be a suitable candidate for isolating endophytic microbiota members that provide plant growth promotion (PGP) and resistance to abiotic stresses. Selected for biocontrol abilities, these endophytes may represent multifaceted and versatile biocontrol agents, combining pathogen biocontrol in addition to PGP and plant protection against abiotic stresses. In this study 117 root culturable bacterial endophytes, including Gram-positive (*Bacillus* and *Brevibacillus*), Gram-negative (*Proteus*, *Providencia*, *Serratia*, *Pantoea*, *Klebsiella*, *Enterobacter* and *Pectobacterium*) and actinomycete *Nocardiopsis* genera have been recovered from *L. monopetalum*. The collection exhibited high levels of biocontrol abilities against bacterial (*Agrobacterium tumefaciens* MAT2 and *Pectobacterium carotovorum* MAT3) and fungal (*Alternaria alternata* XSZJY-1, *Rhizoctonia bataticola* MAT1 and *Fusarium oxysporum* f. sp. radicis lycopersici FORL) pathogens. Several bacteria also showed PGP capacity and resistance to antibiotics and metals. A highly promising candidate *Bacillus licheniformis* LMRE 36 with high PGP, biocontrol, metal and antibiotic, resistance was subsequently tested in planta (potato and olive trees) for biocontrol of a collection of 14 highly damaging *Fusarium* species. LMRE 36 proved very effective against the collection in both species and against an emerging *Fusarium* sp. threatening olive trees culture in nurseries. These findings provide a demonstration of our pyramiding strategy. Our strategy was effective in combining desirable traits in biocontrol agents towards broad-spectrum resistance against pathogens and protection of crops from abiotic stresses. Stacking multiple desirable traits into a single biocontrol agent is achieved by first, careful selection of a host for endophytic microbiota recovery; second, stringent in vitro selection of candidates from the collection; and third, application of the selected biocontrol agents in planta experiments. That pyramiding strategy could be successfully used to mitigate effects of diverse biotic and abiotic stresses on plant growth and productivity. It is anticipated that the strategy will provide a new generation of biocontrol agents by targeting the microbiota of plants in hostile environments.

## 1. Introduction

Plant pathogens can have devastating effects on plant productivity and yield [1,2,3,4,5]. Chemical treatments of pests and pathogens using pesticides in agriculture is still the norm for coping with those threats [6,7,8,9]. Fortunately, growing concern over pesticide impacts on human health, environmental side effects and the spread of pesticide resistance in pathogen populations resulted in stricter legislation over the use of those damaging pesticides [9,10,11,12,13]. This prompted the development of more sustainable approaches mainly based on biocontrol using the plant microbiota [14,15,16,17]. 

The plant microbiota refers to plant associated microflora, including microbes associated to plant surfaces (phyllosphere and rhizosphere) and endophytes thriving inside plant tissues [15,16,18,19,20,21,22]. Endophytic microbiota are being acknowledged for their direct or indirect plant growth and product yield promotion abilities [19,23,24], protection of host plants against phytopathogens [10,11,12,13,25] and improvement of plants’ abilities to withstand environmental biotic and abiotic stresses [19,26].

Recently numerous studies documented that plant metabolites might actually be partially or totally produced by their associated endophytes [13]. In parallel, plant tolerance to biotic and abiotic stresses (e.g., metal stress) has also been attributed partially or totally to their associated microbiota [13]. Therefore, endophytes are believed to produce invaluable potential bioactive secondary metabolites that could be used for the development of new drugs and/or pesticides [16]. They can also be used to promote plant resistance to biotic and abiotic stresses [19]. As a consequence, numerous studies targeted endophytic microbiota characterization form valuable plants, such as medicinal plants and abiotic stress resistant plants [16,18,27,28,29,30,31].

Recent trends in the field of endophyte research implies innovative approaches for their selection. Endophytes are more and more often being screened for multi desirable traits from plants growing under extreme environments [13,15,19,32]. The subsequent use of the endophyte in the field can then equip target plants with new and highly valuable traits, including phytoremediation, biotic and abiotic stresses tolerance and herbicide resistance, among others [33].

The halophyte *Limoniastrum monopetalum*, an evergreen shrub that inhabits marshes, and sandy and rocky soils of coastal environments of the Mediterranean region, have a well-documented phytoremediation potential for the removal of Cd, Zn, Ni and Pb from polluted sites [34,35,36]. It is also considered as a medicinal halophyte, with its leaf and gall infusions used in traditional medicine against human pathogens [37]. Therefore, *L. monopetalum* represents a suitable plant for isolating culturable endophytes that allow not only the biocontrol of pathogens but also may promote plant growth and protect plants against abiotic stresses, such as metal pollution and xenobiotic stress. In this study 117 endophytes have been recovered from *L. monopetalum* and characterized for their plant growth promoting (PGP) abilities, biocontrol of bacterial and fungal pathogens and their resistance to antibiotics and metals. Multifaceted bacterial endophytes have then been selected for efficient PGP, biocontrol and abiotic stress resistance abilities, and shown to be active against a collection of *Fusarium* strains infecting potato and olive trees, as well as an emerging *Fusarium* sp. threatening olive tree production and culture in nurseries.

## 2. Materials and Methods

All chemicals used in this study, unless otherwise indicated, have been provided by Sigma-Aldrich (Buchs, Switzerland).

### 2.1. Sampling

Samples of the *L. monopetalum* plant were collected according to their natural occurrence in a coastal area located at (34°38’21’’N, 10°39’09’’E), Sfax, Tunisia. Ten apparently healthy *L. monopetalum* plants were randomly collected and placed in sterile bags at 4 °C and immediately transported to the laboratory where they were processed immediately.

### 2.2. Inductively Coupled Plasma Optical Emission Spectrometry (ICP-OES) Analysis of Soil Samples

Soil samples of the rhizosphere and in the vicinity of the *L. monopetalum* plants were collected and submitted to ICO-OES analysis. Briefly, the soil samples were dried at 60 °C and ground in fine powder. Elements were than extracted in refluxing with 65% HNO_3_ (Suprapur^®^, Merck KGaA, Germany) for 48 h. After dilution of samples (100 times) by weighing, samples were submitted for ICP-OES analysis. The ICP-OES used for the analyses was an OPTIMA 2100 DV from Perkin Elmer, with an AS-93 plus autosampler. The gases used were argon 5.0, nitrogen 5.0 and compressed air, with the following flow rates: For the plasma 15 mL/min, for auxiliary 0.2 mL/min, for nebulizer 0.65 mL/min.

### 2.3. Isolation of Culturable Endophytic Bacterial Microbiota from L. monopetalum Collected in Tunisia

Roots containing rhizosphere soil were washed with running tap water, disinfected superficially by 70% ethanol for 1 min, rinsed and disinfected superficially with 3% sodium hypochlorite for 20 min. To remove the disinfectant, roots were rinsed three times with sterile double distilled water; samples were then dried with sterile filter paper. To ensure sterilization success, the last washing water was plated on petri plates and the results used as a sterilization control (no colonies were able to grow on the plate after few days). Root tissues were cut into small sections with an average size of 5 mm and were carefully spread on three distinct media and incubated at 28 °C for 5 to 7 days. Morphologically distinct strains of emerging spots or layers from the root pieces were picked for further successive purification. Pure individual colonies were stored in 30% glycerol solution at −20 °C. A total of 117 endophytic bacteria were isolated from the roots of the halophytic *L. monopetalum* plant.

### 2.4. Measurement of PGP Activities of L. monopetalum Bacterial Endophytes

#### 2.4.1. Direct Plant Growth Promoting Rhizobacteria (PGPR) Activities

Five biological repetitions at least have been performed for all direct PGP activities targeted in this study.

##### Growth on Nitrogen Free Medium

Appearance of bacterial growth on nitrogen-free medium containing (g/L): KH_2_PO_4_ (1), MgSO_4_.7H_2_O (0.01), NaCl (0.2), FeSO_4_ (0.005), Mannitol (20), Agar (15), was taken as positive test for the nitrogen fixation experiment [19].

##### Phosphate Solubilization

Phosphate solubilization activity was determined qualitatively by the formation of a clear zone around bacterial growth on Pikovskaya’s agar medium (PVK), containing tricalcium phosphate (Ca_3_HPO_4_) as sole source of phosphate [19].

##### Siderophores Production

Siderophore secretion by the strains was detected using blue agar plates containing the dye Chrome azurol S (Sigma-Aldrich, Buchs, Switzerland). Orange to yellow halos around the bacterial colonies indicated siderophore production [19].

##### Indole Acetic Acid (IAA) Production

For detection of IAA production, isolated colonies were inoculated into Jensen’s agar medium [19] containing (g/L): L-tryptophan (1), Sucrose (20), K_2_HPO_4_ (1), MgSO_4_.7H_2_O (0.5), NaCl (0.5), FeSO_4_ (0.1), Na_2_MoO_4_ (0.005), CaCO_3_ (2) and Agar (15), by the agar well diffusion method. Each well contained 100 µl of overnight bacterial culture. After 48 h of incubation at 28 °C, IAA production was estimated by mixing 20 µL of Salkowsky reagent (2% FeCl_3_ (0.5 M), 35% HClO_4_) with the bacterial culture. Development of a pink color indicated IAA production (the intensity of the color is proportional to the quantitative production of IAA).

#### 2.4.2. Indirect PGP Activities

Indirect PGP activities presented in this study are the average of five biological assays.

##### Screening of Isolates for Extracellular Enzyme Production

Screening of bacterial extracellular enzyme activity was assessed by point inoculation with a bacterial colony on the center of each indicative media plate with a specific substrate. After 24 h of incubation at 28 °C, the halo diameter was estimated by subtracting the colony diameter of the total diameter of clear zones around colonies.

##### Protease Production

Protease activity was checked by measuring resulting clear zone after inoculation of bacterial strains on skimmed milk agar media containing (g/L): Yeast extract (3), casein peptone (5), agar (15) and supplemented after autoclaving with 250 mL of sterile skimmed milk [15].

##### Gelatinase Production

Gelatinase activity was tested according to a slightly modified protocol of Balan et al. [38]. Briefly, bacteria were grown for 24 h at 28 °C on gelatin agar plate containing (g/L): Peptone (4), yeast extract (1), gelatin (15) and agar (15). Gelatin degrading strains develop clear zones around their colonies.

##### Chitinase Production

Chitin agar medium containing (g/L): Colloidal chitin (10), (NH_4_)_2_SO_4_ (2), KH_2_PO_4_ (0.7), MgSO_4_.7H_2_O (0.5), FeSO_4_.7H_2_O (0.01) and agar (15) was used for chitinase screening [19]. Colloidal chitin was prepared following the protocol described by Slama et al. [19]. The clear zone formation around the growing colony was considered an indicator of a positive chitinase activity.

##### Cellulase Production

Isolates were grown on LB medium supplemented with 1% carboxy-methylcellulose (CMC); the revealing of any bacterial activity was done by flooding the plates with Congo red dye 1% and distaining with 1 M NaCl according to the method described in Slama et al. [19]. Cellulase activity was checked by the presence of clear zones surrounding individual isolated colonies.

##### Amylase Production

Amylase producing bacteria were characterized using glucose yeast extract peptone agar (GYP) medium containing (g/L): Glucose (1), yeast extract (0.5), peptone (0.5), agar (15), supplemented with 1% soluble starch. After incubation for 24 h at 28 °C, the plates were flooded with an iodine solution (1% iodine in 2% potassium iodide). The appearance of clear zone around bacterial growth was measured to determine the amylolytic activity [19].

##### Pectinase Production

Pectinolytic activity was determined by growing bacteria in 0.5% pectin Agar medium containing (g/L): pectin (5), yeast extract (1), KH_2_PO_4_ (4), Na_2_HPO_4_ (6) and agar (15). After incubation for 24 h at 28°C, plates were flooded with 1% aqueous solution of Cetyl Trimethyl Ammonium Bromide (CTAB) used as an indicator of pectinolytic activity [19].

##### Glucanase Production

Bacteria were grown on LB medium supplemented with 10 g of barley flour for 24 h at 28 °C. The revealing of any bacterial activity was done by flooding the plates with Congo red dye 1% and distaining with 1M NaCl. The clear zone formed around the bacterial colony indicated a positive glucanase activity.

##### Hydrogen Cyanide (HCN) Production

HCN production by bacterial strains was estimated according to Slama et al. [19] on LB medium supplemented with 4.4 g/L of glycine. The underside of the plate lid includes impregnated Whatman paper with an alkaline picrate solution (2% Na_2_CO_3_, 5% picric acid). After incubation for 48 h at 28°C, production of HCN was indicated by the development of orange to brown color on the Whatman paper.

### 2.5. In Vitro Antibacterial and Antifungal Assays

The antibacterial activity of endophytic strains was tested against two phytopathogenic Gram-negative bacteria: *Agrobacterium tumefaciens* MAT2 and *Pectobacterium carotovorum* MAT3, while antifungal activity was examined using three fungal pathogens: *Alternaria alternata* isolate XSZJY-1 (HQ873733), isolated from pistachios (*Pistacia vera* L.); and *Rhizoctonia bataticola* MAT1 and *Fusarium oxysporum* f. sp. *radicis lycopersici* FORL, isolated from greenhouse tomato roots. One selected strain, *Bacillus licheniformis*, with high antibacterial and antifungal potential, was tested for its antifungal activity against 14 *Fusarium* spp. (isolates Fso10, Fso9, Fso12, Fso11, FCR1, Fso1, Fso5, Fso6, Fso2, Fso3, Fac, Fso13, Fso7 and Fso8) provided by the Olive Tree Institute of Tunisia [39].

### 2.6. Antibacterial Assays

Antibacterial activity was checked by the plate diffusion method [19]. Before incubation, all petri plates were kept at 4 °C for 2 h to enable agar pre-diffusion of produced bacterial metabolites. The plates were then incubated at 28 °C for 24 h and the resultant inhibition growth zone was measured. Controls involved sterile distilled water inoculation.

### 2.7. Antifungal Assays

Bacterial isolates were screened for their in vitro growth inhibition of phytopathogenic fungi by dual culture technique [40]. A pure bacterial colony was streaked 2 cm away from the left side of a 9 cm diameter petri dish containing potato dextrose agar (PDA), and then a 6 mm disc of fungal mycelia was placed on the right side of the petri dish. Plates were incubated at 25 °C until the control (containing only fungal mycelia) reached the plate edge. Mycelial growth inhibition was calculated using the formula: PI = [(C − T)/C] × 100, where C and T represent the colony diameters of fungi on the control and dual culture plates, respectively.

### 2.8. Metal Stress Resistance of L. monopetalum Bacterial Endophytes

The 117 bacterial strains were tested for their ability to grow under different concentrations of five sodium sulfate salt (SO_4_) metals (Cd, Cu, Ni, Zn and Al), at different concentrations: 500, 1000 and 2500 ppm. Bacterial suspension (100 µL) was inoculated on LB broth supplemented with studied metal and incubated at 28 °C for 24 h in a rotatory shaker (120 rpm). Bacterial growth was monitored by measuring the OD at 600 nm using spectrophotometer (Auxilab Zuzi 4251/50, Tunisia) [19].

### 2.9. Antibiotic Resistance of L. monopetalum Bacterial Endophytes

Isolated colonies were checked for their antibiotic resistance against 10 different antibiotics (30 µg/mL): Rifampicin, Tetracycline, Kanamycin, Streptomycin, Cefotaxime, Erythromycin, Amoxicillin, Ampicillin, Penicillin and Ciprofloxacin. Antibiotics were filter sterilized and added aseptically to the LB medium after autoclaving. 5 µL of bacterial suspension was spotted at the surface of the medium and the plate incubated for 24 h at 28 °C. The appearance of bacterial growth indicates resistant bacteria [19].

### 2.10. Bacterial DNA Extraction, 16S-rDNA Amplification, Sequencing and Phylogenetic Analysis

Bacterial genomic DNA extraction and 16S-rDNA amplification have been thoroughly described in Alenezi et al. [10,11]. After purification of amplicons with the Minelute PCR purification kit (Qiagen, Basel, Switzerland) according to the manufacturer’s specifications, purified amplicons were sequenced in both directions using facilities available at the iGE3 (Institute of Genetics and Genomics in Geneva, University of Geneva Medical Center (CMU), Switzerland). Phylogenetic analysis was conducted using procedures described in Mefteh et al. [15].

### 2.11. Isolation of the Emerging PSC1 Olive Tree Fungal Pathogen

Infected olive trees were collected from several nurseries in Tunisia and subjected to pathogen isolation according to Trabelsi et al. [39]. The resulting fungal strain was morphologically identified as *Fusarium* sp. 

### 2.12. In Planta Pathogenicity Assays

Pathogenicity tests were conducted using a collection of 15 isolates of *Fusarium* species (Table S1) and *Fusarium* sp. strain PSC1 able to generate the dieback symptoms on potato tubers and olive twigs. To ascertain *Fusarium* spp. infection, Koch’s postulate was fulfilled in each assay: All pathogens were re-isolated from diseased twigs and tubers, and re-identified morphologically.

### 2.13. Pathogenicity Test on Potato Tubers 

Healthy potato tubers cv. Spunta obtained commercially were used for pathogenicity tests. Potato tubers were washed with running tap water, surface disinfected in 3% solution of commercial bleach for 15 min, rinsed with sterile distilled water (SDW) and air dried under filter-sterilized air flow.

A 5 mm deep plug of external potato tissue was removed from each tuber using a sterilized cork borer. Then, 5 mm diameter mycelial plugs obtained from each of the 15 *Fusarium* spp. were transferred into wounds. Potato tubers with PDA plugs without mycelia, were used as controls. They were placed in sterile plastic bags containing a piece of cotton stuffed with SDW to ensure the maintenance of high humidity, and incubated at 25 °C in darkness for 21 days. The experiment was conducted three times [41].

Penetration diameter was calculated following the formula of Yangui et al. [42]:

P = [W/2 + (D − P)]/2

where: W: Width of the rot (mm); D: Depth of the rot (mm); P: Depth of the well (mm).

### 2.14. Pathogenicity Assays on Olive Twigs

All pathogenicity tests were conducted on olive twigs of 10 cm length collected from healthy olive trees, *Olea europea* cv. Chamlali, growing in a farmland belonging to the olive institute of Sfax (Experimental Unit “Taous”, Olive Institute of Sfax). Olive twigs were washed under running tap water, surface sterilized in 3% solution of commercial bleach for 15 min, rinsed several times with SDW and air dried under filter-sterilized air flow.

Disks (6 mm in diam.) from 2-week-old cultures of *Fusarium* spp. were grown in PDA containing test tubes, then sterile olive twigs were placed in direct contact with them. Disease assessments were made every 10 days, with at least three olive twigs per replicate, by measuring the rot length extension, until reaching complete twig infection. Therefore, evaluations stopped after 35 days. Test tubes with PDA discs without mycelia were used as controls [43].

### 2.15. Effects of LMRE 36 Treatment on Fusarium solani (Fso7) Disease Severity on Potato Tubers

Before inoculation, two holes were made to a depth of 5 mm in each surface sterilized tuber. Inoculation was performed by adding a 5 mm bacterial plug 24 h before or after fungal infection in preventive and curative treatments respectively, while in the concomitant treatment, the antagonistic bacteria and the phytopathogenic fungus were added simultaneously. Non-inoculated tubers were used as controls, tubers infected but not treated were used as negative controls, and treated tubers were used as positive controls. This experiment was conducted 4 times. Penetration diameter was calculated as described previously [42].

### 2.16. Effects of LMRE 36 Treatments on Fusarium solani (Fso6, and Fso7) and Fusarium sp. PSC1 Disease Severity on Olive Twigs

For preventive treatment, surface sterilized olive twigs previously elicited during 24 h in test tubes containing LB media with the antagonistic bacteria *Bacillus licheniformis* LMRE 36 were replaced into test tubes containing PDA (supplemented with antibiotics) inoculated with each *Fusarium* fresh culture.

In the curative treatment, the phytopathogenic fungus *Fusarium* was inoculated in PDA test tube 24 h prior to inoculation with the antagonistic bacteria LMRE 36. The concomitant treatment received the antagonistic bacteria and the phytopathogenic fungus simultaneously. Three controls were applied, water control twigs, infected twigs (*Fusarium* inoculation) and inoculated twigs (LMRE 36 inoculation).

The results were expressed in percentage of inhibition of rot extension; they were calculated according to the present formula:

Inhibition of the extension (%) = [(C − T)/C] × 100

with: C (mm): Inoculated and non-treated twig; T (mm): Infected and treated twig

### 2.17. Statistical Analysis

The statistical analysis of the data was performed using analysis of variance (ANOVA) and, when significant effects were detected, the groups were compared using a post-hoc Tukey’s HSD test. The level of significance used for all statistical tests is 5% (*p* < 0.05). The statistical program used was IBM SPSS Statistics v. 22 (Geneva, Switzerland).

## 3. Results

### 3.1. ICP-OES Analysis of Rhizosphere and Soil Samples Surrounding L. monopetalum

ICP-OES analysis of the rhizosphere and soil samples surrounding *L. monopetalum* proved that soil surrounding *L. monopetalum* was highly rich in sodium, whereas rhizosphere soil has more than six times the reduction of sodium concentration (Table S2). Results also clearly argued the prevalence of deleterious metals, such as Pb, As, Cu and Ni (Table S2).

### 3.2. Isolation of L. monopetalum Bacterial Endophytic Microbiota

**Figure 1:**
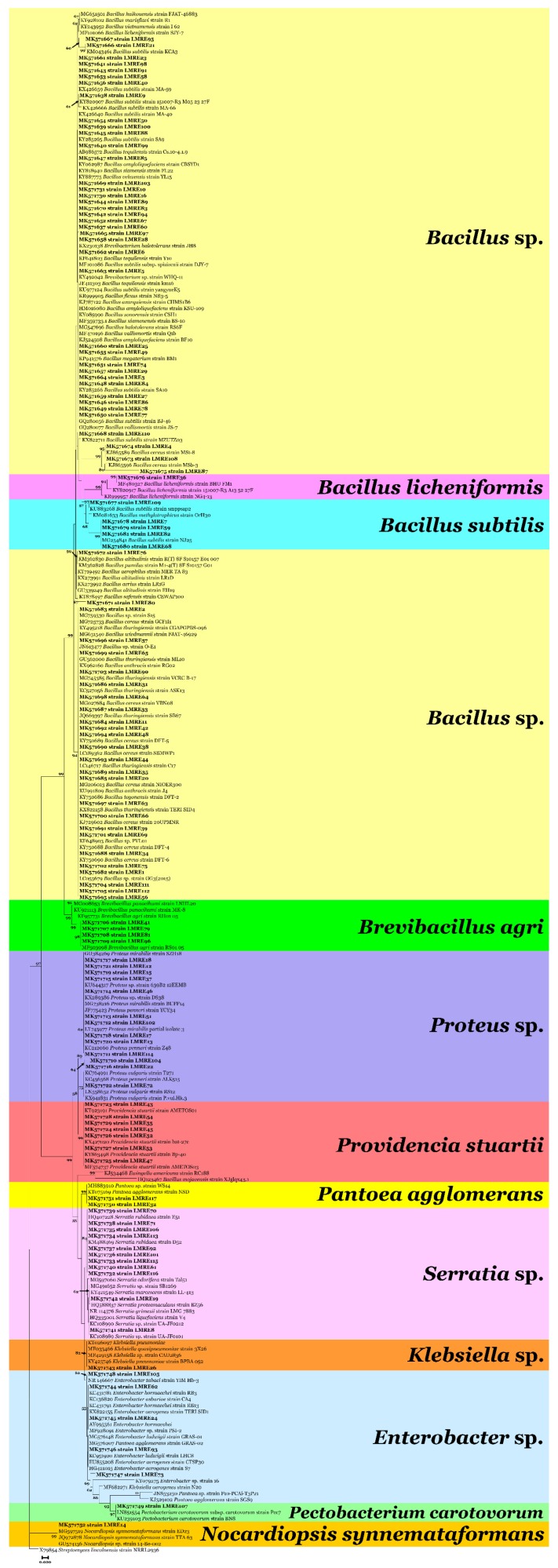
Neighbor-joining phylogenetic tree of Gram-positive and Gram-negative bacteria. The tree was constructed using sequences of 16S-rDNA and rooted to *Streptomyces lincolnensis* strain NRRL2936. Scale bar, number of expected changes per site.

A total of 117 culturable bacterial isolates have been recovered in the time course of this study (Figure 1). Around 65% of them were Gram-positive and belonged to the genera *Bacillus*, *Brevibacillus* and actinomycete genus *Nocardiopsis*. Species diversity within genera was high and reached up to minimum of eight species within the genus *Bacillus*. The remaining 35% of the Gram-negative isolates belonged to the genera *Proteus*, *Providencia*, *Serratia*, *Pantoea*, *Klebsiella*, *Enterobacter* and *Pectobacterium* (Figure 1).

### 3.3. PGP Potential of L. monopetalum Bacterial Endophytes

#### 3.3.1. Direct PGP Activities

**Figure 2:**
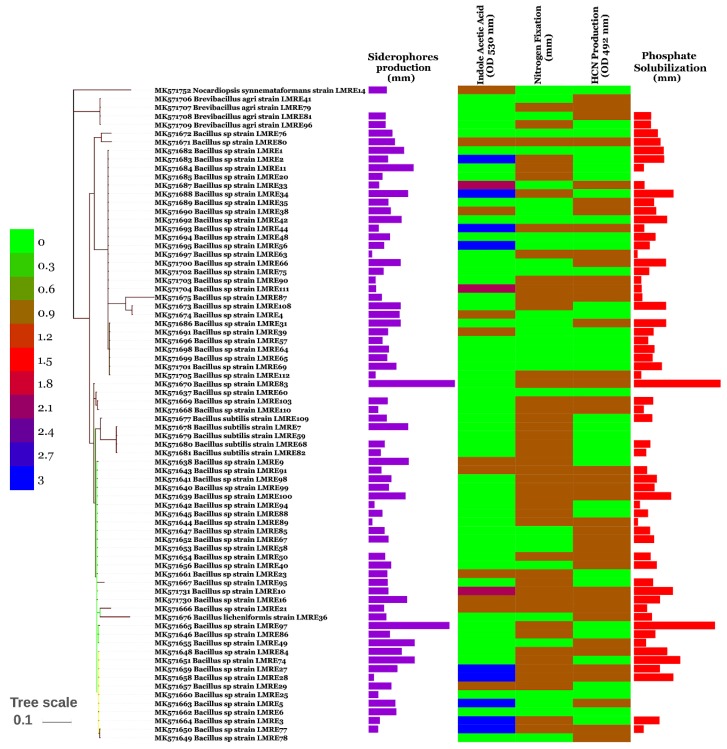
Enzyme activity (IAA production, siderophore production, phosphate solubilization, nitrogen fixation and HCN production) of Gram-positive bacterial isolates.

**Figure 3:**
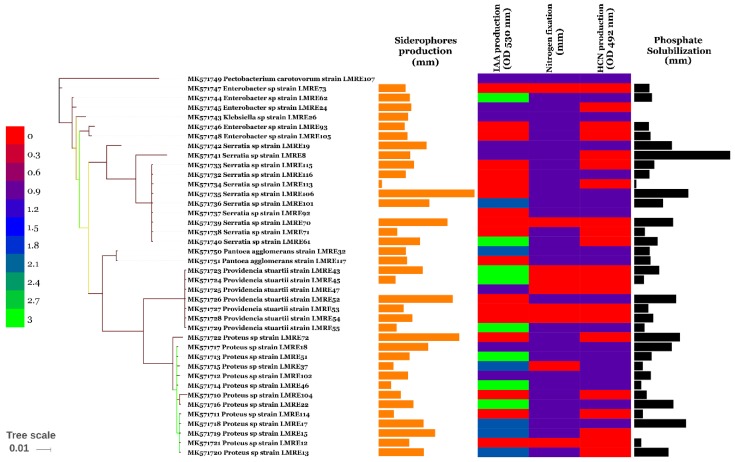
Enzyme activity (IAA production, Siderophore production, Phosphate solubilization, Nitrogen fixation and HCN production) of Gram-negative bacterial isolates.

Direct PGP activities have been performed by screening for growth on nitrogen free medium, phosphate solubilization, siderophores and IAA production. While about half of the total bacteria (49.58%) were able to grow on nitrogen free medium, phosphate solubilization was observed in 52% of the bacterial collection (Figure 2 and Figure 3). *Enterobacter* sp. (LMRE 62) had the maximum phosphate solubilization potential (Figure 3). Siderophore production was prevalent among the microbiome members and only 5% of them lacked siderophore production. Highest siderophore production ability was observed for *Bacillus* sp. isolates LMRE 83 and LMRE 97 with 6.8 mm and 6.4 mm clear zone diameters, respectively. For IAA production seventeen of the 117 isolates (14.52%) had high indole acetic acid activity, 9 (7.69%) had moderate activity and 19 (16.23%) were low IAA producers.

#### 3.3.2. Indirect PGP Activities

**Figure 4:**
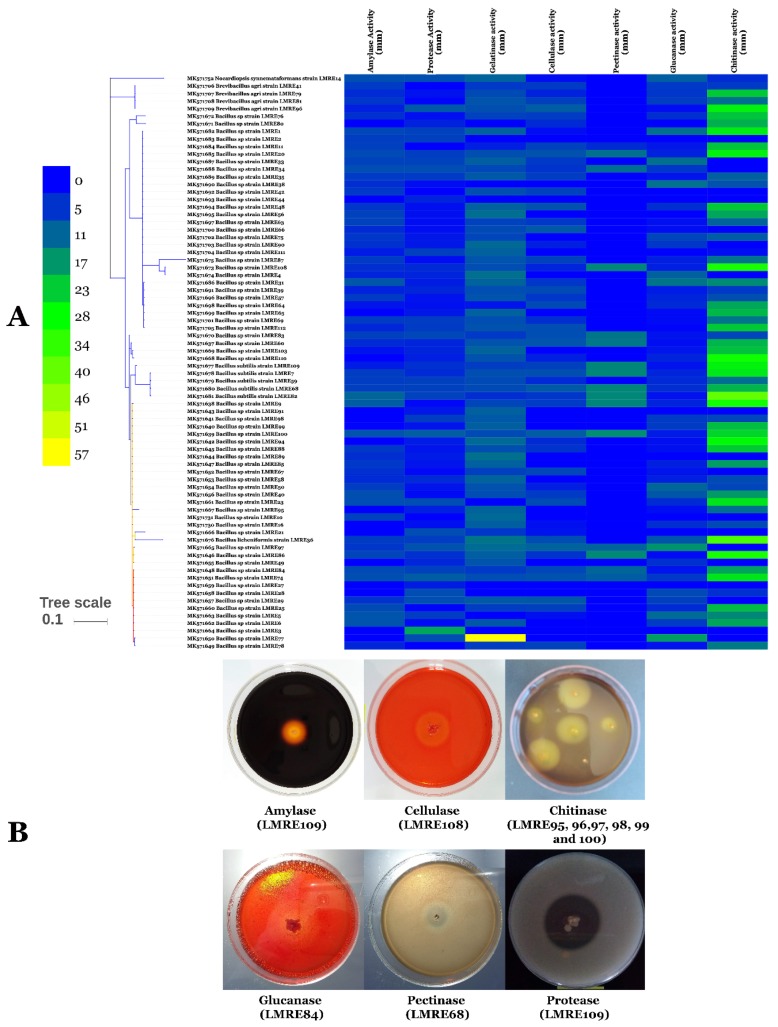
(**A**) Heat-map and (**B**) in vitro extracellular enzyme (amylase, protease, gelatinase, cellulase, pectinase, glucanase and chitinase) activity of Gram-positive bacterial isolates.

**Figure 5:**
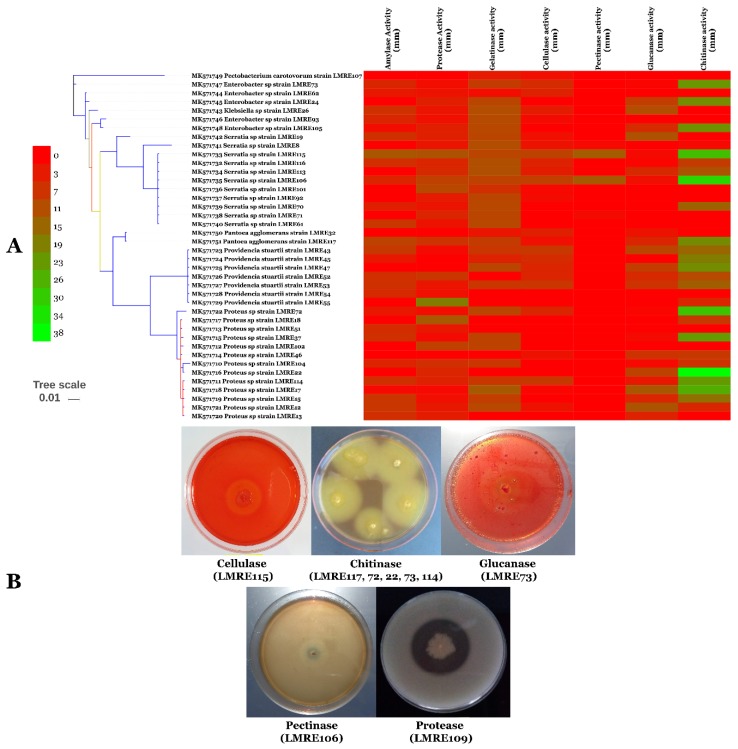
(**A**) Heat-map and (**B**) in vitro extracellular enzyme (amylase, protease, gelatinase, cellulase, pectinase, glucanase and chitinase) activity of Gram-negative bacterial isolates. Numbers indicate degradation halo (mm).

Indirect PGP activities have been checked by screening of microbiome isolates for extracellular enzyme production (protease, gelatinase, chitinase, cellulase, amylase, pectinase and glucanase) and for HCN production. Extracellular protease production was positive for 107 endophytic bacteria and maximum protease activity was recorded in LMRE 30 with halo diameter of 38.12 mm. Gelatinase production was observed in 100 bacterial isolates that were able to degrade gelatin. *Bacillus* sp. LMRE 77 exhibited the highest activity (57.71 mm of halo diameter). Chitinase production was produced by 83 bacterial strains giving chitinolytic zones in plate clearing assay. Among them, strain *Serratia rubidacea* LMRE 106, *Proteus* sp. LMRE22 and 6 *Bacillus* strains (LMRE 9, LMRE 36, LMRE 86, LMRE 82, LMRE 108 and LMRE 110) produced maximum enzymatic activity reaching 40 mm halo diameters. Among the 78 CMC-ase-positive bacteria, 46 were able to produce a clear zone of degradation between 5 and 10 mm of halo diameter. Most of them were identified as *Bacillus* sp. For Amylase production and among the 87 positive strains, peak amylase activity was exhibited by bacterial strain *Serratia rubidacea* LMRE 115 that had a 13.35 mm halo diameter. Twenty isolates out of the collection exhibited pectinase production; among them 17 bacteria gave clear zones of more than 10 mm of halo diameter. Glucanase production was observed in 75% of bacteria tested, with LMRE 77 and LMRE 97 having the highest glucanase activity, with 17.96 mm and 16.82 mm halo diameters, respectively. Hydrogen cyanide production was observed in 49.58% of the bacterial collection (Figure 4A,B and Figure 5A,B).

### 3.4. Biocontrol Ability of L. monopetalum Bacterial Endophytes Towards Relevant Bacterial and Fungal Plant Pathogens

**Figure 6:**
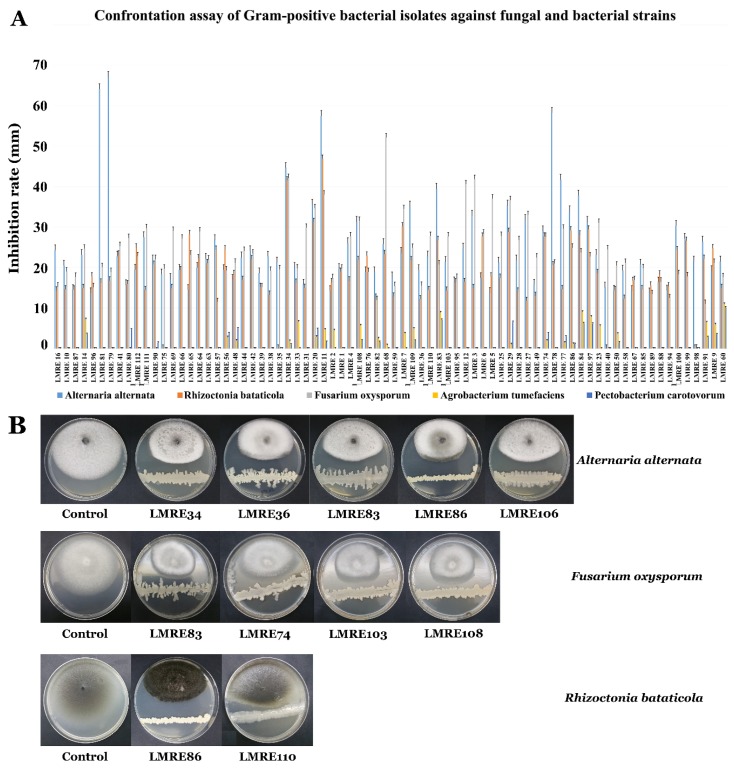
(**A**) Bar-chart of confrontation assay of Gram-positive bacterial isolates against fungal strains and bacterial isolates. (**B**) Confrontation test of *Alternaria alternata* against bacteria.

**Figure 7:**
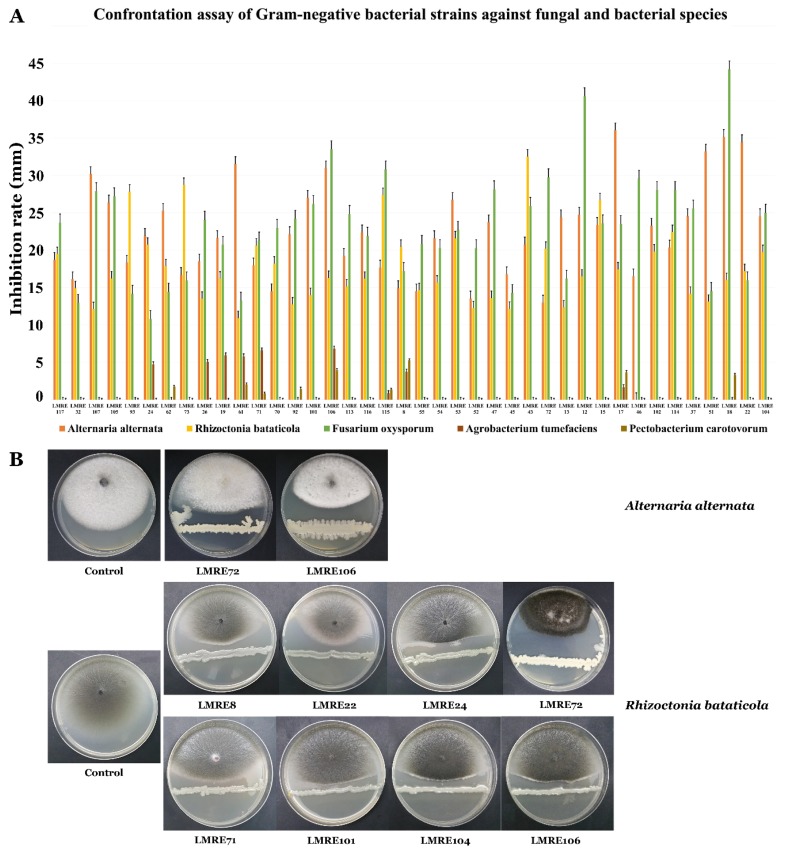
(**A**) Bar-chart of confrontation assay of Gram-negative bacterial isolates against fungal strains and bacterial isolates. (**B**) Confrontation test of *Rhizoctonia bataticola* against bacteria.

Results presented in Figure 6 and Figure 7 for Gram-positive and Gram-negative bacterial isolates clearly attest to high levels of biocontrol abilities of the collection against bacterial and fungal pathogens tested. 78 bacterial isolates proved effective in inhibiting both types of phytopathogenic bacteria. Endophytic strain *Bacillus subtilis* LMRE 60 for instance, had the highest antibacterial activity against both of the phytopathogenic bacteria *Agrobacterium tumefaciens* MAT2 and *Pectobacterium carotovorum* MAT3, with 11 and 10-mm inhibitory diameters, respectively (Figure 6A,B and Figure 7A,B). Against the three distinct fungal pathogens tested; *Alternaria alternata* isolate XSZJY-1, *Rhizoctonia bataticola* MAT1 and *Fusarium oxysporum* f. sp. *radicis lycopersici* (FORL); *L. monopetalum* bacterial endophytes exhibited high levels of biocontrol abilities. In fact, the 117 bacterial isolates were able to inhibit *A. alternata* isolate XSZJY-1 fungal growth with different percentages, with the maximum percentage of inhibition being 67.15% registered for *Brevibacillus* sp. LMRE 79 had minimal inhibition of 13% for *Proteus vulgaris* LMRE 72. *Bacillus* sp. LMRE 11 and LMRE 34 had the ability to inhibit both fungal isolates *A. alternata* and *R. bataticola* with up to 40% inhibition. *Bacillus subtilis* LMRE 68 caused maximum growth inhibition of the highly damaging plant pathogen *F. oxysporum* f. sp. *radicis lycopersici*, FORL (52.17%).

### 3.5. Resistance of L. monopetalum Bacterial Endophytic Communities to Antibiotics and Metals

**Figure 8:**
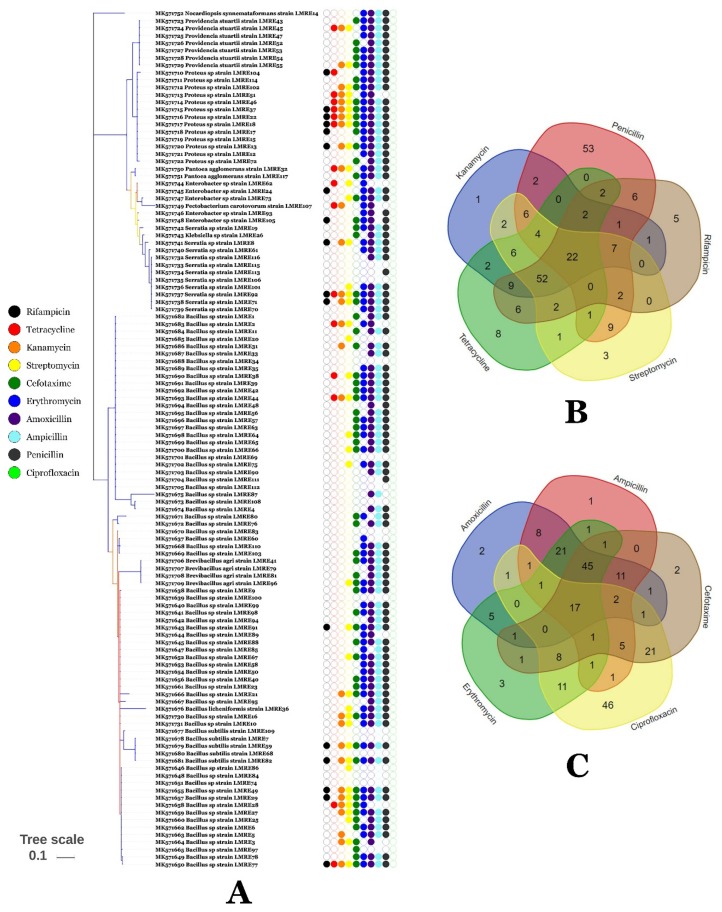
(**A**) Shape-plot and (**B**,**C**) Venn diagram of antibiotic resistance of Gram-positive and Gram-negative bacterial isolates.

Analysis of antibiotic resistance profiles of the *L. monopetalum* bacterial endophytic microbiota showed high levels of resistance to numerous antibiotics tested (Figure 8A–C). While high levels of resistance were observed towards penicillin, ampicillin, amoxicillin and erythromycin, few resistant bacteria had resistance to rifampicin, tetracycline, kanamycin and streptomycin. Up to 22 and 17 bacteria out of the root endophyte community were resistant to the five antibiotics (kanamycin, penicillin, rifampicin, streptomycin and tetracycline) and (amoxicillin, ampicillin, cefotaxime, ciprofloxacin and erythromycin), respectively (Figure 8B,C).

**Figure 9:**
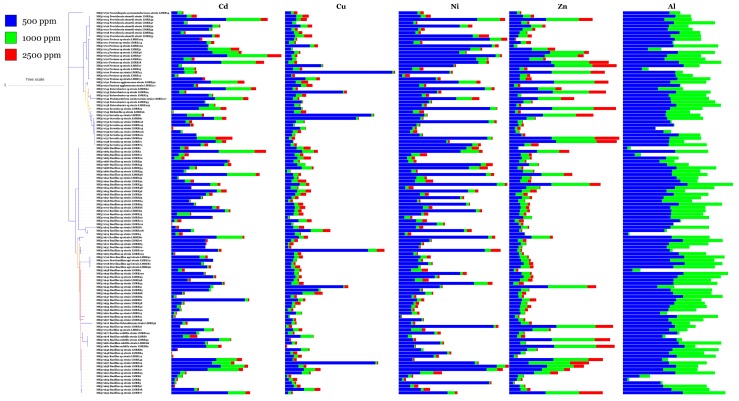
Multi-value bar-charts of heavy metal (Cd, Cu, Ni, Zn and Al) resistance of Gram-positive and Gram-negative bacterial isolates. (Dilutions of heavy metals: 500 ppm, 1000 ppm and 2500 ppm).

Qualitative assay of bacterial resistance to metal stress showed that the bacteria were able to survive in the presence of several increasing concentrations of heavy metals. The first studied heavy metal concentration was 500 ppm, in which, 33 bacterial strains were able to resist all five heavy metals. The highest number of metal tolerant bacteria (115) has been commonly recorded for Aluminum (Al_2_(SO_4_)_3_) followed by Zinc (ZnSO_4_), Nickel (NiSO_4_) and Cadmium (CdSO_4_) with 110, 106 and 95 resistant bacteria respectively (Figure 9). Copper (CuSO_4_) was the most damaging metal, against whom we found only 46 resistant bacteria. Increasing the concentration of all metals up to 1000 ppm resulted in only two *Bacillus* sp. (LMRE 2 and LMRE 27) that were able to resist all metals. As the heavy metal concentrations increased (2500 ppm) the number of resistant bacterial isolates decreased, falling to zero resistant bacteria in Copper (CuSO_4_) containing media (Figure 9).

### 3.6. Biocontrol Ability of L. monopetalum Bacterial Endophyte LMRE 36 Towards Fusarium spp. Plant Pathogens

**Figure 10:**
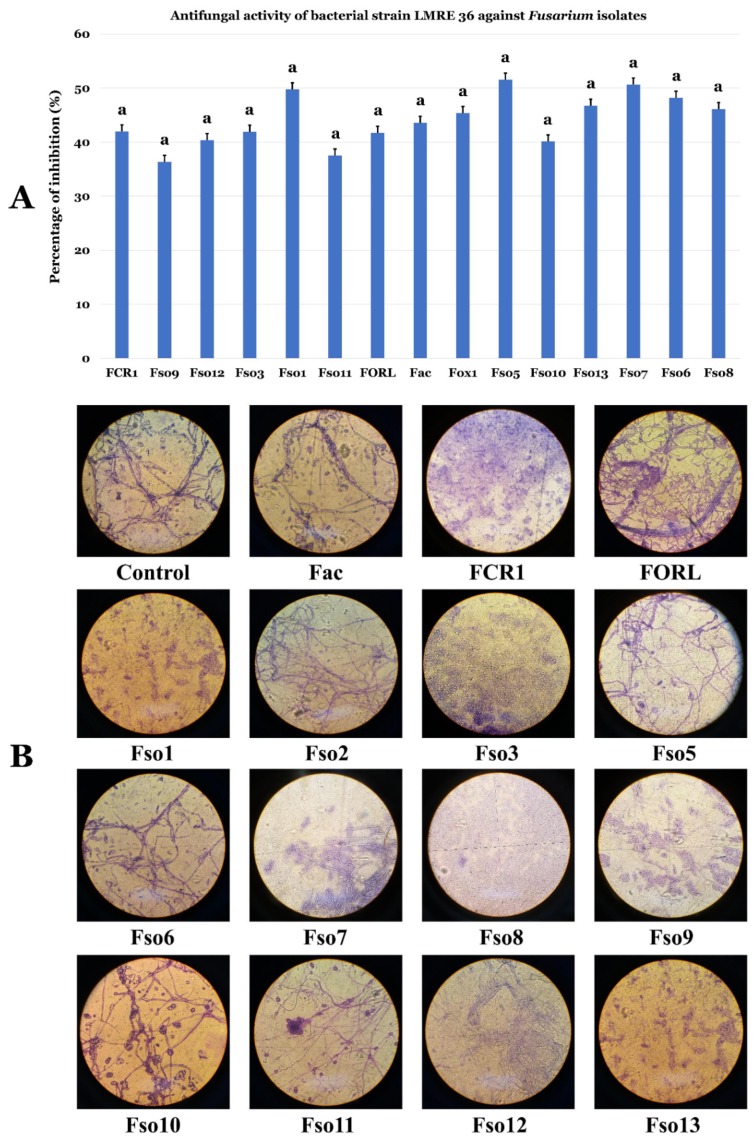
(**A**) Antifungal activity of bacterial strain LMRE 36 against *Fusarium* isolates. Data presents mean ± standard error. Bars labelled with the same letters were not significantly different among the treatments at *p* < 0.05 using Tukey’s HSD test. (**B**) Microscopical confrontation assay of LMRE36 against different *Fusarium* strains.

*Bacillus licheniformis* LMRE 36 strain was able to inhibit a collection of 14 phytopathogenic *Fusarium* spp. with up to 35% percentage of inhibition (Figure 10).

#### 3.6.1. Effects of LMRE 36 Treatments on *F. solani* Fso7 Disease Severity on Potato Tubers

**Figure 11:**
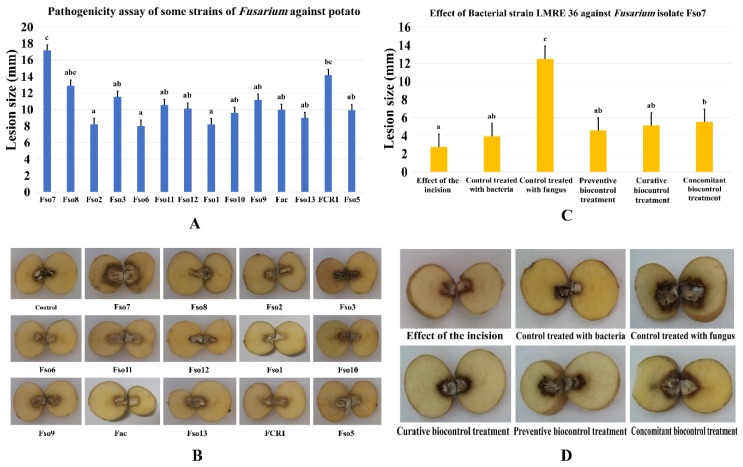
(**A**,**B**) Pathogenicity assay of *Fusarium* spp. strains against potato tubers. (**C**) Effects of LMRE 36 bacterial isolate on *Fusarium solani* strain Fso7 on potato tubers rot severity as compared to controls. (**D**) Effect of preventive, concomitant and curative biocontrol treatment on potato tubers at 10, 22 and 35-days post-inoculation with *Fusarium solani* strain Fso7. Data presents mean ± standard error. Bars labelled with different letters are significantly different among the treatments at *p* < 0.05 using Tukey’s HSD test.

A collection of 14 *Fusarium* strains collected from infected olive trees orchards where numerous vegetables are also being planted has been tested for their pathogenicity towards potato tubers. After 3 weeks of incubation all *Fusarium* species proved effective in developing rot around the inoculation site (Figure 11A,B). While, inoculated tubers with *Fusarium solani* Fso7 reached the highest penetration average (17.13 mm), *Fusarium* spp. FCR1, Fso8, Fso3, Fso9, Fso11 and Fso12 had average penetration rates of 14.15 mm, 12.88 mm, 11.52 mm, 11.17 mm, 10.53 mm and 10.11 mm respectively. All remaining fungi had less than a 10 mm penetration average. The use of antagonistic bacterium LMRE 36 in controlling the *Fusarium solani* infection proved effective in controlling disease severity. Thus, significant reductions in decay severity were obtained with the three types of tested biological treatments, with close percentages: 70%, 67% and 66% for preventive, curative and concomitant treatments, respectively, in comparison with the negative control inoculated only by *F. solani* Fso7 (Figure 11C,D).

#### 3.6.2. Effects of LMRE 36 Treatments on *F. solani* Fso7 Disease Severity on Olive Twigs

**Figure 12:**
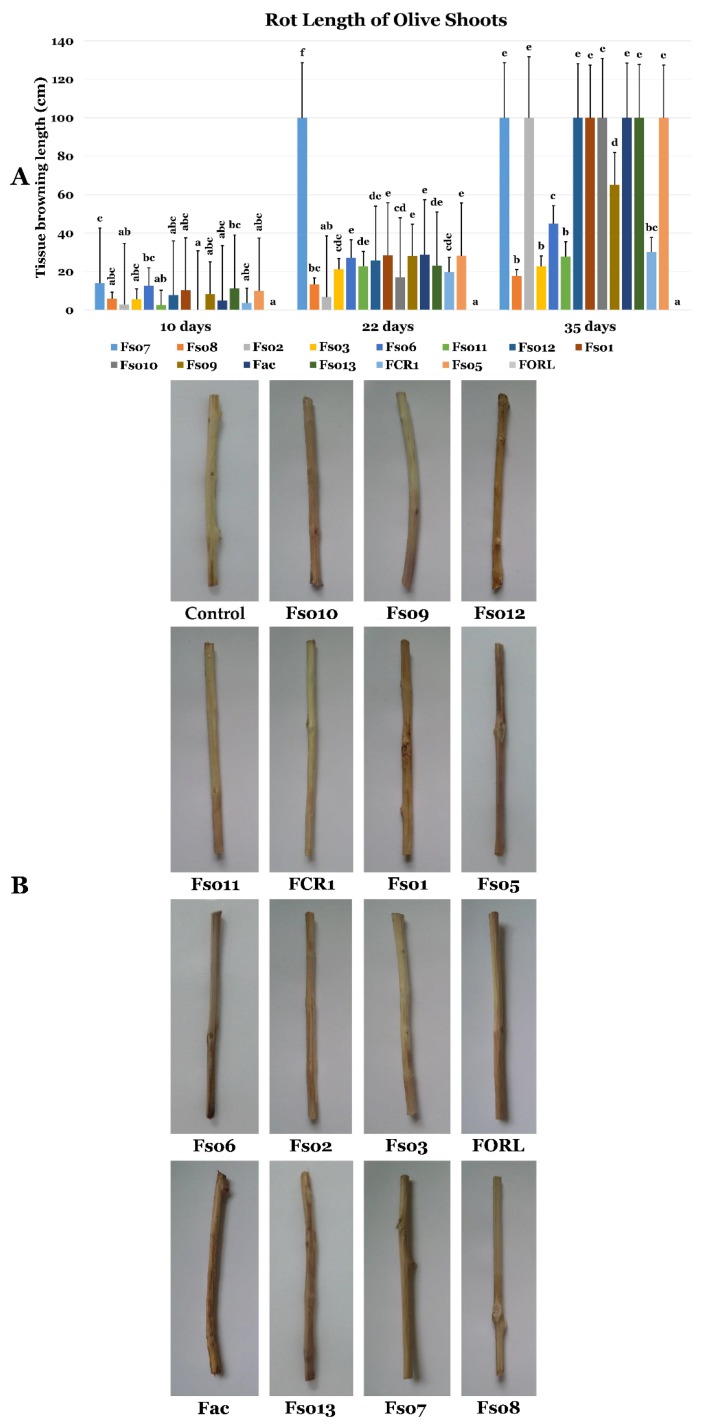
(**A**,**B**) Rot length of olive tree shoots (mm) caused by *Fusarium* spp. strains at 10, 22 and 35-days post-inoculation. Data presents mean ± standard error. Bars labelled with different letters are significantly different among the treatments at *p* < 0.05 using Tukey’s HSD test.

Using the 14 *Fusarium* strains, collection for inoculation of olive twigs proved that *Fusarium* disease symptoms on olive twigs appeared from the first 10 days with varying disease severity, when compared to control olive twigs. In fact, after 10 days, only *Fusarium solani* isolates (Fso7, Fso6, Fso1 and Fso13) exceeded 10 cm of tissue browning, indicating infection progression. While after one month, *Fusarium solani* isolates in addition to other isolates of *F. oxysporum* and *F. acuminatum* induced total rot of olive twigs (Fso7, Fso6, Fso1, Fso13, Fso2, Fso12, Fso10, Fac, Fso13 and Fso5, Figure 12). 

**Figure 13:**
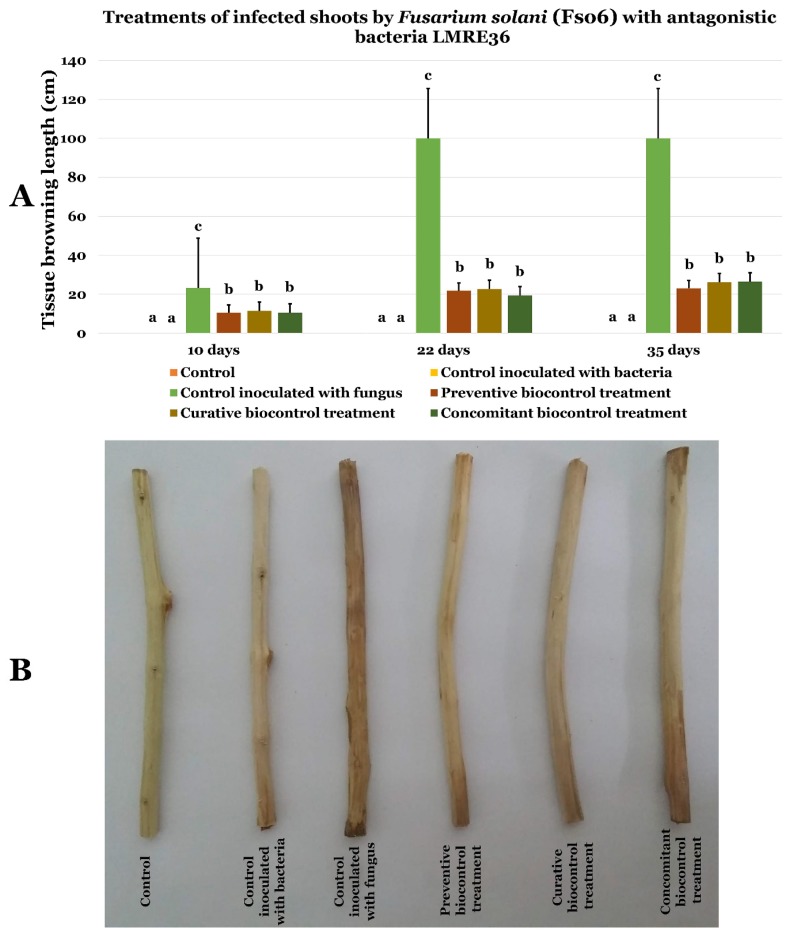
(**A**) Effects of LMRE 36 bacterial isolates on *Fusarium solani* strain Fso6 on olive tree shoots rot severity as compared to controls. (**B**) Effect of preventive, concomitant, and curative biocontrol treatment on olive tree shoots at 10, 22 and 35-days post-inoculation with *Fusarium solani* strain Fso6. Data presents mean ± standard error. Bars labelled with different letters are significantly different among the treatments at *p* < 0.05 using Tukey’s HSD test.

**Figure 14:**
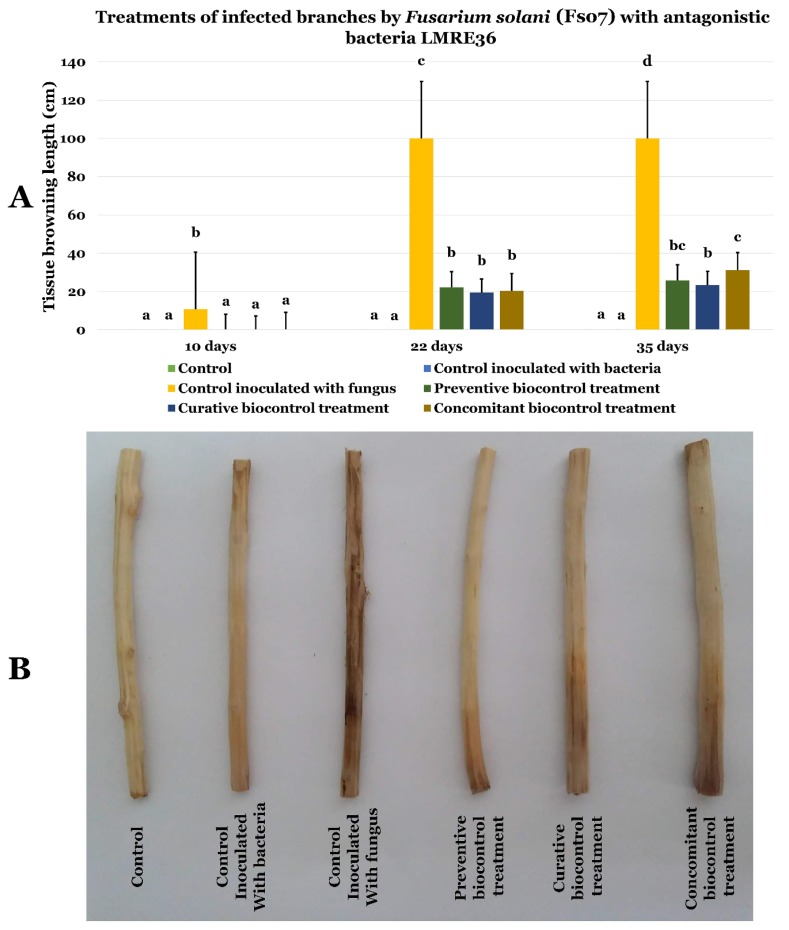
(**A**) Effects of LMRE 36 bacterial isolates on *Fusarium solani* strain Fso7 on olive tree shoots rot severity as compared to controls. (**B**) Effect of preventive, concomitant, and curative biocontrol treatment on olive tree shoots at 10, 22 and 35-days post-inoculation with *Fusarium solani* strain Fso7. Data presents mean ± standard error. Bars labelled with different letters are significantly different among the treatments at *p* < 0.05 using Tukey’s HSD test.

Using *Bacillus licheniformis* LMRE 36 in preventive, curative and concomitant biocontrol assays on olive twigs inoculated with *Fusarium solani* (Fso6, and Fso7) showed clear protection against disease progression (Figure 13 and Figure 14, respectively). Indeed, when the negative control (inoculated with *Fusarium* spp. and not treated with LMRE 36) was completely rotten (10 cm) all bacterial treatments inhibited the *Fusarium* spp. progression up to 75% compared to the control.

### 3.7. Isolation of the Emerging PSC1 Olive Tree Fungal Pathogen

PSC1 was isolated from diseased olive trees produced in nurseries. Morphological analysis suggested the fungal pathogen to belong to *Fusarium* genus. Koch’s postulates confirmed that PSC1 was responsible for the observed mortality of olive trees in nurseries (Figure S1).

### 3.8. Biocontrol Ability and Effects on PSC1 Disease Severity on Olive Twigs of L. monopetalum Bacterial Endophyte LMRE 36 Towards PSC1

**Figure 15:**
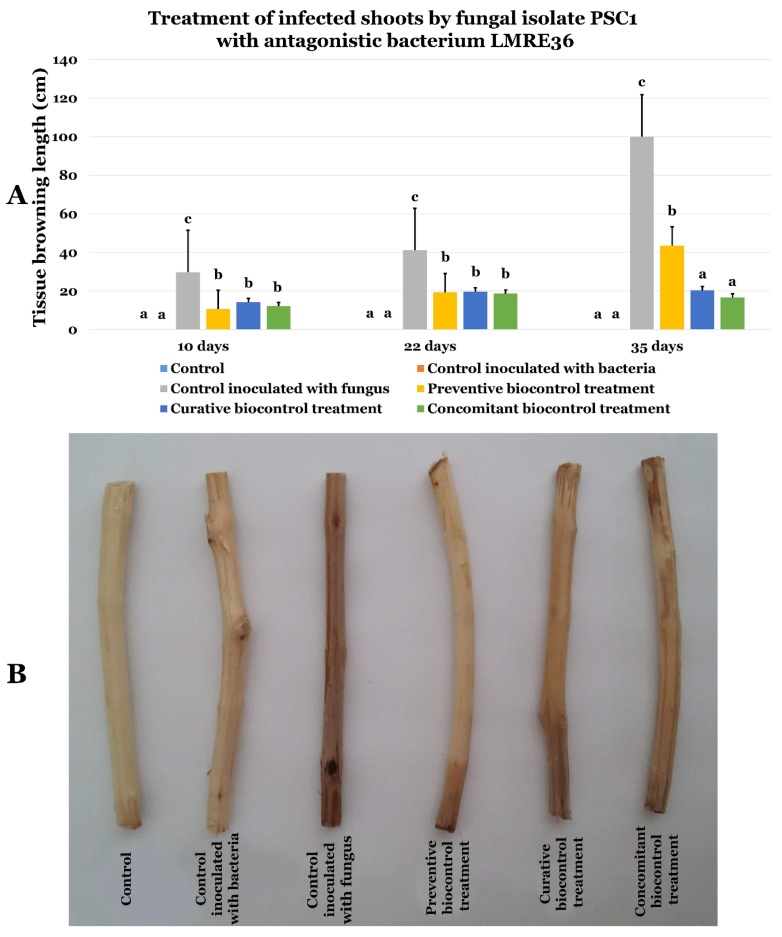
(**A**) Effects of LMRE 36 bacterial isolate on *Fusarium solani* strain PSC1 on olive tree shoots rot severity as compared to controls. (**B**) Effect of preventive, concomitant, and curative biocontrol treatment on olive tree shoots at 10, 22 and 35-days post-inoculation with *Fusarium solani* strain PSC1. Data presents mean ± standard error. Bars labelled with different letters are significantly different among the treatments at *p* < 0.05 using Tukey’s HSD test.

*Bacillus licheniformis* LMRE 36 proved very effective in inhibiting PSC1 growth in vitro with inhibition rates of more than 50% (Figure S1). Using LMRE 36 in preventive, curative and concomitant biocontrol assays on olive twigs inoculated with *Fusarium sp.* PSC1 showed clear protection against disease progression (Figure 15). Indeed, when the negative control (inoculated with *Fusarium* sp. and not treated with LMRE 36) was completely rotten (10 cm) all bacterium treatments inhibited the *Fusarium* spp. progression up to 75% compared to the control.

## 4. Discussion

Coastal salt marshes are considered special environments, and are one of the most biologically productive habitats on Earth [44]. This ecosystem is inhabited by a variety of halophytic plants that constitute a source of various stress resistances. Halophytic plant *L. monopetalum* (L.) Boiss., which is an ever-green dwarf shrub Plumbaginaceae, is very common in marshes of coastal environments and saltworks of the Mediterranean region and south Portugal [28,29]. The plant has a well-documented phytoremediation potential, such as the removal of Cd, Zn, Ni and Pb from polluted sites [28,29,30]. It also contains various chemical compounds of medicinal use. Generally, halophytes with traditional health benefits in folk medicine are a source of attraction for modern comprehensive microbial and chemical investigations [38]. All these conditions make unusual extremophilic associated microbiotas, having unique mechanisms of coping with extreme environments, capable of producing unusual metabolites [19,45,46,47]. Plant growth promoting endophytic bacteria represent a widely studied group; they use different mechanisms to penetrate into the plant tissues, particularly in roots which represent the most common mode of entry of endophytic bacteria into their host plant [48].

Consequently, intimate and non-harmful associations can be formed between bacteria and their host plants [49]. This is the biggest difference between endophytes and plant growth promoting rhizobacteria (PGPR) [13]. Like rhizobacteria, there are several mechanisms by which endophytic bacteria offer several benefits to their host plants, particularly by growth promotion, and protection from, and under biotic and abiotic stresses [50]. The variation of endophytic communities between various plants depend on many variables, such as plant tissue analyzed, plant growth stage, plant health, nutritional state, biotic and abiotic stresses [15,21,51]. ICP-OES analysis of the rhizosphere and soil surrounding *L. monopetalum* proved that that sodium concentrations were strongly reduced in the rhizosphere compared to surrounding soil reflecting; therefore, the importance of *L. monopetalum* cannot be understated in terms of coping with harmful salt and other stresses. Based on those facts, we aimed for the first time to isolate, screen, characterize and identify endophytic bacteria from *L. monopetalum* plant roots. 16S–rDNA phylogenetic analysis was used to characterize our endophytic bacterial collection. The results demonstrated that they belong to 10 different genera; the most abundant was *Bacillus* genus (61%), followed by *Proteus* (11%), *Serratia* (9%), *Providencia* (6%), Enterobacter (4%), *Brevibacillus* (3%) and *Pantoea* (2%). The last 3 genera: *Klebsiella*, *Pectobacterium* and *Nocardiopsis* had equal percentages: 1% for each one. Our findings were in agreement with Passari et al. [52] who also found *Bacillus* as the dominant endophytic genus in roots of *Clerodendrum colebrookianum* Walp. and *Coriandrum sativum* respectively [52]. Several other reports documented our reported genera from various crops: As an example, endophytes *Bacillus licheniformis*, *Klebsiella* sp., *Pantoea* sp., *Bacillus* sp. and *Bacillus cereus* isolated from maize roots [51], *Bacillus* sp., and *Enterobacter* sp., isolated from corn roots [53] and *Serratia plymuthica* strain G3 isolated from wheat stems [54].

The culturable collection was then evaluated for its PGP potential by the analysis of direct and indirect PGP capacities. Direct PGP activities have been performed by screening for growth on nitrogen free medium, phosphate solubilization, siderophores and IAA production. Half of the isolated bacteria were able to express nitrogenase. The most productive genera were *Bacillus*, *Proteus* and *Serratia*. Similar results have been reported in Ji et al. [55] where *Bacillus* species have been identified as diazotrophic bacteria. Rice seeds treated with these bacteria showed improved growth, increases in height and dry weight, and antagonistic effects against pathogenic fungi [55]. Similarly, Hongrittipun et al. [56] recovered numerous nitrogen-fixing bacteria from roots and leaves of rice.

As phosphorus mostly occurs in the soil in an insoluble form [13], the intervention of phosphate solubilizing endophytic bacteria becomes important for plants. In our study, *Enterobacter* sp. was the best phosphate producer in comparison to other bacteria. Similarly, Delgado et al. [57] proved the ability of endophytic *Enterobacter* sp. to solubilize phosphate on a large scale [57]. Iron is also an essential component for plant growth, given that most of it exists in highly insoluble ferric (Fe^3+^) form in soils (unavailable for plant uptake). Under iron-limiting conditions PGP bacteria produce low-molecular-weight compounds called siderophores to competitively acquire ferric ions [13]. In our study, 108 endophytic bacterial isolates were able to produce siderophores with *Bacillus* sp. strains being the best producers. Our results are in agreement with those of Wani and Khan [58] where *Bacillus* sp. PSB10 was identified as a siderophore producer [58]. Additionally, halotolerant *Bacillus* bacteria isolated from host plant *Arthrocnemum macrostachyum* [59] and from *Psoralea corylifolia* L. [60] possessed multiple growth promoting abilities, including siderophore production.

Microorganisms have the ability to synthesize indole acetic acid (IAA), a vital plant growth regulator hormone, through various pathways utilizing tryptophan as main precursor [13]. In this study, we found that IAA production was carried out by 45 bacterial strains from different genera like *Bacillus* sp., *Proteus* sp., *Serratia* sp., *Providencia* sp., *Enterobacter* sp., *Klebsiella* sp., *Pontoea* sp., *Pectobacterium carotovorum* and *Nocardiopsis* sp. It is well documented that a vast majority of endophytic bacteria synthesized IAA [13].

Indirect PGP activities have been checked by screening isolates for extracellular enzyme production (protease, gelatinase, chitinase, cellulase, amylase, pectinase and glucanase) and for HCN production. Microorganisms with extracellular enzyme activities are not only helpful in organic matter decomposition and PGP, but also play an important role in the disease suppression by inhibiting soil borne pathogens [61,62]. Our findings revealed that 12 endophytic bacteria were able to produce all seven tested enzymes (Protease, Gelatinase, Chitinase, Cellulase, Amylase, Pectinase and Glucanase). 

Among those culturable isolates, we found 10 *Bacillus* strains, including sic *Bacillus subtilis*, two *Bacillus* spp. and two *Bacillus ceureus*, together with two *Serratia rubidaea* isolates. Those results are comparable to those of El-Deeb et al. [63] who demonstrated that *Bacillus* sp., *Bacillus pumilus*, *Bacillus licheniformis* and *Bacillus megaterium* have multiple enzyme production capacities. Moreover, Petersen and Tisa [64] proved that secretion of potent lytic enzymes, especially chitinases, is characteristic of most *Serratia* species. In a recent study [65] it was reported that extracellular lytic enzyme activities, including cellulase, pectinase and protease were displayed by numerous cucurbit seed-associated bacterial endophytes. Generally, hydrolytic enzymes of endophytes may help intracellular colonization and development of endophytism [13].

Another important attribute of PGP bacterial microbiota was the production of HCN which plays an important role in disease suppression of crop plants. In the current study about half of the microbiota bacterial isolates (49.58%) were effective HCN producers, most of them (62%) belong to the genus *Bacillus*, similar to results reported by Belbahri et al. [13].

Endophytic bacteria recovered in this study were all tested for their antagonistic activity against two bacterial and three fungal phytopathogens. Results clearly showed that a collection of 78 bacterial isolates were able to inhibit both phytopathogenic bacteria *Agrobacterium tumefaciens* MAT2 and *Pectobacterium carotovorum* MAT3. Those findings are in agreement with several studies showing the potential of bacterial endophytes as biocontrol agents [53,66,67]. The highest antagonistic inhibitory potential against all bacterial and fungal phytopathogens was recorded in *Bacillus* genera with maximum growth inhibition of 52% against *Fusarium oxysporum*, and up to 40% against *Alternaria alternata* XSZJY-1 and *Rhizoctonia bataticola* MAT1. Among them, *B. licheniformis* LMRE 36 was able to inhibit 14 phytopathogenic *Fusarium* spp. for up to 35%. Multiple reports demonstrated the capacities of *Bacillus* strains to control *Fusarium*-induced plant diseases [68]. The antagonistic capacities of endophytic bacteria are explained by their plant growth promoting abilities, both directly and indirectly during normal and stress conditions [13]. 

The *Limoniastrum monopetalum* endophytic community displayed also high levels of multi-resistance to diverse antibiotics with up to 39 bacteria resistant to five antibiotics. That suggests high a capacity of the endophytic bacteria to cope with xenobiotics that could interfere with plant growth and development. Heavy metals negatively influence endophytic bacterial diversity and composition in plants [69,70]. The hyper-accumulator plants constitute a complex and specialized endophytic bacterial flora with high levels of resistance to heavy metals [69,70]. That may be due to the adaptation strategies of endophytic bacteria to heavy metal containing environment [13].

Those findings are in agreement with this study where our isolates were able to resist all five metals tested in the following order: CuSO_4_ > NiSO_4_ > CdSO_4_ > ZnSO_4_ > Al_2_(SO_4_)_3_. At 500 ppm heavy metal concentration, more than 100 bacteria were able to resist to Aluminum, Zinc and Nickel. At 1000 ppm only 2 *Bacillus* spp. were able to resist all metals. Those findings are in agreement with Shin et al. [69] who reported that endophytic bacterial strain *Bacillus* sp. MN3-4 isolated from the roots of the metal hyperaccumulator plant *Alnus firma* had evolved a better-defined metal-resistant mechanism. Ma et al. [70], demonstrated that *Bacillus thuringiensis* GDB-1 and *Bacillus pumilus* E2S2 isolated from *Alnus firma* and *Sedum* respectively, were able to resist at least three heavy metals (Cd, Cu and Zn) [70]. Lastly, we found that copper (CuSO_4_) was the most damaging metal where none of the isolated bacteria were able to resist to 2500 ppm.

Plant fungal pathogens could be responsible for massive destruction of crop yield [1]. Particularly, pathogenic soil borne *Fusarium* species represents one of the most threatening diseases affecting multiple plant crops, such as olive trees (*Olea europaea* L.) and potato plants (*Solanum tuberosum* L.) [39]. In our study, using a collection of 15 *Fusarium* spp. isolated from diseased olive trees intercropped with solanaceous crops (potato, pepper, tomato, etc.), we were able to confirm their pathogenicity on olive twigs. *Fusarium solani* isolates, including Fso7, Fso6, Fso1, Fso13, Fso12, Fso10 and Fso5, *Fusarium oxysporum* Fso2, and *Fusarium acuminatum* Fac were able to induce brown wilt symptoms on the longitudinal sections of olive twigs (*Olea europaea* L. cv. ‘Chemlali’), until reaching total rot of the twigs. That *Fusarium* spp. pathogenic collection was also tested on potato tubers (*Solanum tuberosum* L. cv; ‘Spunta’). The results showed that *Fusarium solani* Fso7 caused the highest dieback symptoms.

Using *Bacillus licheniformis* LMRE 36 in *Fusarium* in planta biocontrol experiments proved that preventive, curative and concomitant treatment results on both olive twigs and potato tubers were very efficient in coping with the development of the infection by the most infectious *Fusarium solani* isolates Fso7 and Fso6. Percentage of decay severity reduction reached at least 66%, suggesting high efficiency of biocontrol ability of LMRE 36 bacterial species. Multiple other reports confirmed the *Bacillus* strains’ capabilities to overcome *Fusarium* spp. diseases [19,71,72,73]. The basis for the observed activity of *Bacillus* spp. against *Fusarium* pathogenic species has been linked to secondary metabolites that strongly interfere with fungal pathogens [74,75]. To test the validity of our strategy against emerging pathogens, we isolated *Fusarium* sp. PSC1 an emerging pathogen of olive tree production and growth in nurseries. LMRE 36 bacterial endophyte proved active against PSC1 in in vitro confrontation testing and in planta preventive, curative and concomitant treatments.

## 5. Conclusions

Our strategy proved effective in pyramiding desirable traits in biocontrol agents, for achieving broad-spectrum resistance against several pathogens and demonstrating crop protection from common abiotic stresses. Stacking multiple desirable traits into single biocontrol agent could, therefore, be generalized and can be achieved by first, careful selection of host for endophyte recovery; second, stringent in vitro selection of putative candidates from the collection; and third, application of the selected biocontrol agents in in planta experiments. This pyramiding strategy could be successfully used in future studies to mitigate the effects of diverse biotic and abiotic stresses on plant growth and productivity. It is anticipated that this strategy will provide a new generation biocontrol agents in future studies targeting highly biotic and abiotic stress resistant plants thriving in hostile environments.

## Supplementary Materials

The following are available online at https://www.mdpi.com/2076-2607/7/8/249/s1, Figure S1: (A) Infected olive tree (right) in comparison to healthy one (left); (B) microscopical confrontation assay of bacterial isolate LMRE 36 against *Fusarium* isolates. Table S1: Details of fungal species used in this study. Table S2: ICP-OES analysis for soil and rhizosphere around the *LM* plants.

## References

[1] Lara E., Belbahri L. (2011). SSU rRNA reveals major trends in oomycete evolution. Fungal Divers..

[2] Olson A., Aerts A., Asiegbu F., Belbahri L., Bouzid O., Broberg A., Canbäck B., Coutinho P.M., Cullen D., Dalman K. (2012). Insight into trade-off between wood decay and parasitism from the genome of a fungal forest pathogen. New Phytol..

[3] Luchi N., Ghelardini L., Belbahri L., Quartier M., Santini A. (2013). Rapid detection of *Ceratocystis platani* inoculum by quantitative Real-Time PCR assay. Appl. Environ. Microbiol..

[4] Prospero S., Vercauteren A., Heungens K., Belbahri L., Rigling D. (2013). *Phytophthora* diversity and the population structure of *Phytophthora ramorum* in Swiss ornamental nurseries. Plant Pathol..

[5] Abad Z.G., Abad J.A., Cunnington J.H., Smith I.W., Blomquist C., Balci Y., Moralejo E., Perez-Sierra A., Abad-Campos P., Alvarez-Bernaola L.A. (2014). *Phytophthora niederhauserii* sp. nov. a new polyphagous species mostly isolated from ornamentals potted plants in twelve countries of five continents. Mycologia.

[6] Alenezi F.N., Weitz H.J., Belbahri L., Nidhal J., Luptáková L., Jaspars M., Woodward S. (2015). Draft Genome Sequence of *Aneurinibacillus migulanus* NCTC 7096. Genome Announc..

[7] Alenezi F.N., Weitz H.J., Belbahri L., Ben Rebah H., Luptakova L., Jaspars M., Woodward S. (2015). Draft genome sequence of *Aneurinibacillus migulanus* strain Nagano. Genome Announc..

[8] Belbahri L., Alenezi F.N., Luptakova L., Rateb M.E., Woodward S. (2015). Complete genome sequence of *Aneurinibacillus migulanus* E1, a gramicidin S and d-phenylalanyl-l-propyl diketopiperazine-deficient mutant. Genome Announc..

[9] Cherrad S., Charnay A., Hernandez C., Steva H., Belbahri L., Vacher S. (2018). Emergence of boscalid-resistant strains of *Erysiphe necator* in French vineyards. Microbiol. Res..

[10] Alenezi F.N., Fraser S., Belka M., Dogmuş T.H., Heckova Z., Oskay F., Belbahri L., Woodward S. (2016). Biological control of *Dothistroma* needle blight on pine with *Aneurinibacillus migulanus*. For. Pathol..

[11] Alenezi F.N., Rekik I., Bełka M., Ibrahim A.F., Luptakova L., Jaspars M., Woodward S., Belbahri L. (2016). Strain-level diversity of secondary metabolism in the biocontrol species *Aneurinibacillus migulanus*. Microbiol. Res..

[12] Alenezi F.N., Rekik I., Chenari Bouket A., Luptakova L., Weitz H.J., Rateb M.E., Jaspars M., Woodward S., Belbahri L. (2017). Increased biological activity of *Aneurinibacillus migulanus* strains correlates with the production of new gramicidin secondary metabolites. Front. Microbiol..

[13] Belbahri L., Chenari Bouket A., Rekik I., Alenezi F.N., Vallat A., Luptakova L., Petrovova E., Oszako T., Cherrad S., Vacher S. (2017). Comparative genomics of *Bacillus amyloliquefaciens* strains reveals a core genome with traits for habitat adaptation and a secondary metabolites rich accessory genome. Front. Microbiol..

[14] Rodriguez R.J., Henson J., van Volkenburgh E., Hoy M., Wright L., Beckwith F., Kim Y.O., Redman R.S. (2008). Stress tolerance in plants via habitat-adapted symbiosis. ISME J..

[15] Mefteh F.B., Daoud A., Chenari Bouket A., Alenezi F.N., Luptakova L., Rateb M.E., Kadri A., Gharsallah N., Belbahri L. (2017). Fungal root microbiome from healthy and brittle leaf diseased date palm trees (*Phoenix dactylifera* L.) reveals a hidden untapped arsenal of antibacterial and broad spectrum antifungal secondary metabolites. Front. Microbiol..

[16] Mefteh F.B., Daoud A., Chenari Bouket A., Thissera B., Kadri Y., Cherif-Silini H., Eshelli M., Alenezi F.N., Vallat A., Oszako T. (2018). Date palm trees root-derived endophytes as fungal cell factories for diverse bioactive metabolites. Int. J. Mol. Sci..

[17] Orozco-Mosqueda M.D.C., Rocha-Granados M.D.C., Glick B.R., Santoyo G. (2018). Microbiome engineering to improve biocontrol and plant growth-promoting mechanisms. Microbiol. Res..

[18] Strobel G. (2018). The emergence of endophytic microbes and their biological promise. J. Fungi.

[19] Slama H., Cherif-Silini H., Chenari Bouket A., Qader M., Silini A., Yahiaoui B., Alenezi F.N., Luptakova L., Triki M.A., Vallat A. (2019). Screening for *Fusarium* antagonistic bacteria from contrasting niches designated the endophyte *Bacillus halotolerans* as plant warden against *Fusarium*. Front. Microbiol..

[20] Compant S., Samad A., Faist H., Sessitsch A. (2019). A review on the plant microbiome: Ecology, functions, and emerging trends in microbial application. J. Adv. Res..

[21] Kandel S.L., Joubert P.M., Doty L.S. (2017). Bacterial endophyte colonization and distribution within plants. Microorganisms.

[22] Sessitsch A., Pfaffenbichler N., Mitter B. (2019). Microbiome applications from lab to field: Facing complexity. Trends Plant Sci..

[23] Vurukonda S.S.K.P., Giovanardi D., Stefani E. (2018). Plant growth promoting and biocontrol activity of *Streptomyces* spp. as endophytes. Int. J. Mol. Sci..

[24] Zhou L.S., Tang K., Guo S.X. (2018). The plant growth-promoting fungus (PGPF) *Alternaria* sp. A13 markedly enhances *Salvia miltiorrhiza* root growth and active ingredient accumulation under greenhouse and field conditions. Int. J. Mol. Sci..

[25] Compant S., Duffy B., Nowak J., Clément C., Barka E.A. (2005). Use of plant growth-promoting bacteria for biocontrol of plant diseases: Principles, mechanisms of action, and future prospects. Appl. Environ. Microbiol..

[26] He A.L., Niu S.Q., Zhao Q., Li Y.S., Gou J.Y., Gao H.J., Suo S.Z., Zhang J.L. (2018). Induced salt tolerance of perennial ryegrass by a novel bacterium strain from the rhizosphere of a desert shrub *Haloxylon ammodendron*. Int. J. Mol. Sci..

[27] Abdennabi R., Bardaa S., Mehdi M., Rateb M.E., Raab A., Alenezi F.N., Sahnoun Z., Gharsallah N., Belbahri L. (2016). *Phoenix dactylifera* L. sap enhances wound healing in Wistar rats: Phytochemical and histological assessment. Int. J. Biol. Macromol..

[28] Li P., Wu Z., Liu T., Wang Y. (2016). Biodiversity, phylogeny, and antifungal functions of endophytic fungi associated with *Zanthoxylum bungeanum*. Int. J. Mol. Sci..

[29] Daoud A., Ben Mefteh F., Mnafgui K., Turki M., Jmal S., Ben Amar R., Ayadi F., El-Feki A., Abid L., Rateb M.E. (2017). Cardiopreventive effect of ethanolic extract of date palm pollen against isoproterenol induced myocardial infarction in rats through the inhibition of the angiotensin-converting enzyme. Exp. Toxicol. Pathol..

[30] Sánchez-López A.S., Pintelon I., Stevens V., Imperato V., Timmermans J.P., González-Chávez C., Carrillo-González R., Van Hamme J., Vangronsveld J., Thijs S. (2018). Seed endophyte microbiome of *Crotalaria pumila* unpeeled: Identification of plant-beneficial methylobacteria. Int. J. Mol. Sci..

[31] Prieto P., Schilirò E., Maldonado-González M., Valderrama R., Barroso-Albarracín J.B., Mercado-Blanco J. (2011). Root hairs play a key role in the endophytic colonization of olive roots by *Pseudomonas* spp. with biocontrol activity. Microb. Ecol..

[32] Hardoim P.R., van Overbeek L.S., Berg G., Pirttilä A.M., Compant S., Campisano A., Döring M., Sessitsch A. (2015). The hidden world within plants: Ecological and evolutionary considerations for defining functioning of microbial endophytes. Microbiol. Mol. Biol. Rev..

[33] Yadav A., Yadav K. (2017). Exploring the potential of endophytes in agriculture: A minireview. Adv. Plants Agric. Res..

[34] Cambrolle J., Mancilla-Leytón J.M., Muñoz-Vallés S., Figueroa-Luque E., Luque T., Figueroa M.E. (2013). Evaluation of zinc tolerance and accumulation potential of the coastal shrub *Limoniastrum monopetalum* (L.) Boiss. Environ. Exp. Bot..

[35] Cambrolle J., Mancilla-Leytón J.M., Muñoz-Vallés S., Figueroa-Luque E., Luque T., Figueroa M.E. (2013). Effects of copper sulfate on growth and physiological responses of *Limoniastrum monopetalum*. Environ. Sci. Pollut. Res..

[36] Manousaki E., Galanaki K., Papadimitriou L., Kalogerakis N. (2014). Metal phytoremediation by the halophyte *Limoniastrum monopetalum* (L.) Boiss: Two contrasting ecotypes. Int. J. Phytoremediat..

[37] Trabelsi N., Falleh H., Jallali I., Daly A.B., Hajlaoui H., Smaoui A., Abdelly C., Ksouri R. (2012). Variation of phenolic composition and biological activities in *Limoniastrum monopetalum* L. organs. Acta Physiol. Plant..

[38] Balan S.S., Nethaji R., Sankar S., Jayalakshmi S. (2012). Production of gelatinase enzyme from *Bacillus* spp. isolated from the sediment sample of Porto Novo Coastal sites. Asian Pac. J. Trop. Biomed..

[39] Trabelsi R., Sellami H., Gharbi Y., Krid S., Cheffi M., Kammoun S., Dammak M., Mseddi A., Gdoura R., Triki M.A. (2017). Morphological and molecular characterization of *Fusarium* spp. associated with olive trees dieback in Tunisia. 3 Biotech..

[40] Bibi F., Strobel G.A., Naseer M.I., Yasir M., Khalaf Al-Ghamdi A.A., Azhar E.I. (2018). Microbial flora associated with the halophyte *Salsola imbricate* and its biotechnical potential. Front. Microbiol..

[41] Hortova B., Novotny D., Erban T. (2014). Physiological characteristics and pathogenicity of eight *Neofabraea* isolates from apples in Czechia. Eur. J. Hortic. Sci..

[42] Yangui T., Sayadi S., Dhouib A. (2013). Sensitivity of *Pectobacterium carotovorum* to hydroxytyrosol-rich extracts and their effect on the development of soft rot in potato tubers during storage. Crop. Prot..

[43] Romero J., Raya M.C., Roca L.F., Agustí-Brisach C., Moral J., Trapero A. (2018). Phenotypic, molecular and pathogenic characterization of *Phlyctema vagabunda*, causal agent of olive leprosy. Plant Pathol..

[44] Gong Y., Bai J.L., Yang H.T., Zhang W.D., Xiong Y.W., Ding P., Qin S. (2018). Phylogenetic diversity and investigation of plant growth-promoting traits of actinobacteria in coastal salt marsh plant rhizospheres from Jiangsu, China. Syst. Appl. Microbiol..

[45] Qin Y., Druzhinina I.S., Pan X., Yuan Z. (2016). Microbially mediated plant salt tolerance and microbiome-based solutions for saline agriculture. Biotechnol. Adv..

[46] Qin S., Li W.J., Dastager S.G., Hozzein W.N. (2016). Actinobacteria in special and extreme habitats: Diversity, function roles, and environmental adaptations. Front. Microbiol..

[47] Spence C., Alff E., Johnson C., Ramos C., Donofrio N., Sundaresan V., Bais H. (2014). Natural rice rhizospheric microbes suppress rice blast infections. BMC Plant Biol..

[48] Passari A.K., Mishra V.K., Saikia R., Gupta V.K., Singh B.P. (2015). Isolation, abundance and phylogenetic affiliation of endophytic actinomycetes associated with medicinal plants and screening for their in vitro antimicrobial biosynthetic potential. Front. Microbiol..

[49] Timmusk S., Behers L., Muthoni J., Muraya A., Aronsson A.C. (2017). Perspectives and challenges of microbial application for crop improvement. Front. Plant Sci..

[50] Khan N., Martínez-Hidalgo P., Ice T.A., Maymon M., Humm E.A., Nejat N., Sanders E.R., Kaplan D., Hirsch A.M. (2018). Antifungal activity of *Bacillus* species against *Fusarium* and analysis of the potential mechanisms used in biocontrol. Front. Microbiol..

[51] Marag P.S., Suman A. (2018). Growth stage and tissue specific colonization of endophytic bacteria having plant growth promoting traits in hybrid and composite maize (*Zea mays* L.). Microbiol. Res..

[52] Passari A.K., Mishra V.K., Leo V.V., Gupta V.K., Singh B.P. (2016). Phytohormone production endowed with antagonistic potential and plant growth promoting abilities of culturable endophytic bacteria isolated from *Clerodendrum colebrookianum* Walp. Microbiol. Res..

[53] Szilagyi-Zecchin V.J., Ikeda A.C., Hungria M., Adamoski D., Kava-Cordeiro V., Glienke C., Galli-Terasawa L.V. (2014). Identification and characterization of endophytic bacteria from corn (*Zea mays* L.) roots with biotechnological potential in agriculture. AMB Express.

[54] Liu X., Yu X., Yang Y., Heeb S., Gao S., Chan K.G., Camara M., Gao K. (2018). Functional identification of the prnABCD operon and its regulation in *Serratia plymuthica*. Appl. Microbiol. Biotechnol..

[55] Ji S.H., Gururani M.A., Chun S.C. (2014). Isolation and characterization of plant growth promoting endophytic diazotrophic bacteria from Korean rice cultivars. Microbiol. Res..

[56] Hongrittipun P., Youpensuk S., Rerkasem B. (2014). Screening of nitrogen fixing endophytic bacteria in *Oryza sativa* L.. J. Agric. Sci..

[57] Delgado M., Mendez J., Rodriìguez-Herrera R., Aguilar C.N., Cruz-Hernaìndez M., Balagurusamy N. (2014). Characterization of phosphate solubilizing bacteria isolated from the arid soils of a semi-desert region of north-east Mexico. Biol. Agric. Hortic..

[58] Wani P.A., Khan M.S. (2010). *Bacillus* species enhance growth parameters of chickpea (*Cicer arietinum* L.) in chromium stressed soils. Food Chem. Toxicol..

[59] Navarro-Torre S., Barcia-Piedras J.M., Mateos-Naranjo E., Redondo-Gómez S., Camacho M., Caviedes M.A., Pajuelo E., Rodríguez-Llorente I.D. (2017). Assessing the role of endophytic bacteria in the halophyte *Arthrocnemum macrostachyum* salt tolerance. Plant Biol..

[60] Sorty A.M., Meena K.K., Choudhary K., Bitla U.M., Minhas P.S., Krishnani K.K. (2016). Effect of plant growth promoting bacteria associated with halophytic weed (*Psoralea corylifolia* L.) on germination and seedling growth of wheat under saline conditions. Appl. Biochem. Biotechnol..

[61] Zhao S., Zhou N., Zhao Z.Y., Zhang K., Wu G.H., Tian C.Y. (2016). Isolation of endophytic plant growth–promoting bacteria associated with the halophyte *Salicornia europaea* and evaluation of their promoting activity under salt stress. Curr. Microbiol..

[62] Fernando W.G.D., Ramarathnam R.K.A.S., Savchuk S.C. (2005). Identification and use of potential bacterial organic antifungal volatiles in biocontrol. Soil Biol. Biochem..

[63] El-Deeb B., Fayez K., Gherbawy Y. (2013). Isolation and characterization of endophytic bacteria from *Plectranthus tenuiflorus* medicinal plant in Saudi Arabia desert and their antimicrobial activities. J. Plant Interact..

[64] Petersen L.M., Tisa L.S. (2013). Friend or foe? A review of the mechanisms that drive *Serratia* towards diverse lifestyles. Can. J. Microbiol..

[65] Khalaf E.M., Raizada M.N. (2016). Taxonomic and functional diversity of cultured seed associated microbes of the cucurbit family. BMC Microbiol..

[66] Sandhya V., Shrivastava M., Ali S.Z., Sai Shiva Krishna Prasad V. (2017). Endophytes from maize with plant growth promotion and biocontrol activity under drought stress. Russ. Agric. Sci..

[67] White J.F., Kingsley K.I., Kowalski K.P., Irizarry I., Micci A., Soares M.A., Bergen M.S. (2018). Disease protection and allelopathic interactions of seed-transmitted endophytic Pseudomonads of invasive seed grass (*Phragmites australis*). Plant Soil.

[68] Raza W., Ling N., Zhang R., Huang Q., Xu Y., Shen Q. (2017). Success evaluation of the biological control of *Fusarium* wilts of cucumber, banana, and tomato since 2000 and future research strategies. Crit. Rev. Biotechnol..

[69] Shin M.N., Shim J., You Y., Myung H., Bang K.S., Cho M., Oh B.T. (2012). Characterization of lead resistant endophytic *Bacillus* sp. MN3-4 and its potential for promoting lead accumulation in metal hyperaccumulator *Alnus firma*. J. Hazard. Mater..

[70] Ma Y., Oliveira R.S., Nai F., Rajkumar M., Luo Y., Rocha I., Freitas H. (2015). The hyperaccumulator *Sedum plumbizincicola* harbors metal-resistant endophytic bacteria that improve its phytoextraction capacity in multi-metal contaminated soil. J. Environ. Manag..

[71] Gond S.K., Bergen M., Torres M.S., White J.F. (2015). Effect of bacterial endophyte on expression of defense genes in Indian popcorn against *Fusarium moniliforme*. Symbiosis.

[72] Jeong M.H., Lee Y.S., Cho J.Y., Ahn Y.S., Moon J.H., Hyun H.N., Cha G.S., Kim K.Y. (2017). Isolation and characterization of metabolites from *Bacillus licheniformis* MH48 with antifungal activity against plant pathogens. Microb Pathog..

[73] Nigris S., Baldan E., Tondello A., Zanella F., Vitulo N., Favaro G., Guidolin V., Bordin N., Telatin A., Barizza E. (2018). Biocontrol traits of *Bacillus licheniformis* GL174, a culturable endophyte of *Vitis vinifera* cv. Glera. BMC Microbiol..

[74] Santhanam R., Menezes R.C., Grabe V., Li D., Baldwin I.T., Groten K. (2019). A suite of complementary biocontrol traits allows a native consortium of root-associated bacteria to protect their host plant from a fungal sudden-wilt disease. Mol. Ecol..

[75] Kejela T., Thakkar V.R., Thakor P. (2016). *Bacillus* species (BT42) isolated from *Coffea arabica* L. rhizosphere antagonizes *Colletotrichum gloeosporioides* and *Fusarium oxysporum* and also exhibits multiple plant growth promoting activity. BMC Microbiol..

