# Supplementary material for: Screening of the High-Rhizosphere Competent Limoniastrum monopetalum’ Culturable Endophyte Microbiota Allows the Recovery of Multifaceted and Versatile Biocontrol Agents
Source: Microorganisms. 2019 Aug 9;7(8):249. doi: 10.3390/microorganisms7080249 (PMC6723025; doi:10.3390/microorganisms7080249)
Supplement: Supplementary file 1 [file microorganisms-07-00249-s001.zip › microorganisms-543210-supplementary/Table S1.docx]

Table S1. Details of fungal species used in this study.

|  | Species | Strain | Source | Country | GenBank Accession |
| --- | --- | --- | --- | --- | --- |
|  |  |  |  |  | ITS |
| 1 | *Fusarium solani* | Fso3 | *Olea europaea* | Tunisia | KU528851 |
| 2 | *Fusarium solani* | Fso12 | *Olea europaea* | Tunisia | KU528862 |
| 3 | *Fusarium solani* | Fso11 | *Olea europaea* | Tunisia | KU528861 |
| 4 | *Fusarium solani* | Fso7 | *Olea europaea* | Tunisia | KU528857 |
| 5 | *Fusarium solani* | Fso5 | *Olea europaea* | Tunisia | KU528854 |
| 6 | *Fusarium solani* | Fso6 | *Olea europaea* | Tunisia | KU528855 |
| 7 | *Fusarium solani* | Fso8 | *Olea europaea* | Tunisia | KU528858 |
| 8 | *Fusarium solani* | Fso9 | *Olea europaea* | Tunisia | KU528859 |
| 9 | *Fusarium solani* | Fso1 | *Olea europaea* | Tunisia | KU528848 |
| 10 | *Fusarium solani* | Fso10 | *Olea europaea* | Tunisia | KU528860 |
| 11 | *Fusarium solani* | Fso13 | *Olea europaea* | Tunisia | KU528863 |
| 12 | *Fusarium solani* | Fso2 | *Olea europaea* | Tunisia | KU528850 |
| 13 | *Fusarium oxysporum* | Fox1 | *Olea europaea* | Tunisia | KU528844 |
| 14 | *Fusarium oxysporum* f.sp. *radices lycopersicum* | FORL | Tomato | Tunisia | - |
| 15 | *Fusarium acuminatum* | Fac | *Olea europaea* | Tunisia | KU528866 |
| 16 | *Fusarium chlamydosporum* | FCR1 | *Olea europaea* | Tunisia | KU528845 |
| 17 | *Fusarium* sp. | PSC1 | *Olea europaea* | Tunisia | - |
| 18 | *Alternaria alternata* | XSZJY-1 | *Pistacia vera* | Tunisia | HQ873733 |
| 19 | *Rhizoctonia bataticola* | MAT1 | *Olea europaea* | Tunisia | - |
