# Supplementary material for: Screening of the High-Rhizosphere Competent Limoniastrum monopetalum’ Culturable Endophyte Microbiota Allows the Recovery of Multifaceted and Versatile Biocontrol Agents
Source: Microorganisms. 2019 Aug 9;7(8):249. doi: 10.3390/microorganisms7080249 (PMC6723025; doi:10.3390/microorganisms7080249)
Supplement: Supplementary file 1 [file microorganisms-07-00249-s001.zip › microorganisms-543210-supplementary/Table S2.docx]

Table S2. ICP-OES analysis for soil and rhizosphere around the *LM* plants.

| Elements | Soil (mg/Kg) | Rhizosphere (mg/Kg) |
| --- | --- | --- |
| Cd | 0 | 0 |
| Pb | 3.597 | 3.168 |
| As | 2.353 | 2.617 |
| Hg | 0 | 0 |
| Co | 0 | 1.515 |
| Ni | 3.736 | 3.443 |
| Cu | 3.597 | 3.306 |
| Al | 4611.335 | 5869.265 |
| Ba | 56.448 | 53.854 |
| Cr | 9.131 | 8.815 |
| Fe | 3833.684 | 4650.511 |
| Mn | 52.160 | 53.991 |
| Mo | 0 | 0 |
| Zn | 11.483 | 11.845 |
| Se | 0 | 0 |
| Rb | 8.578 | 9.504 |
| Sn | 0 | 0 |
| Sr | 259.217 | 240.544 |
| Li | 21.306 | 16.252 |
| Ag | 0 | 0 |
| Na | 7326.294 | 1154.610 |
| Mg | 8144.874 | 7825.687 |
| K | 1337.014 | 1491.371 |
| Ca | 32388.493 | 23396.879 |
